# β_2_-Microglobulin Amyloid Fibril-Induced Membrane Disruption Is Enhanced by Endosomal Lipids and Acidic pH

**DOI:** 10.1371/journal.pone.0104492

**Published:** 2014-08-06

**Authors:** Sophia C. Goodchild, Tania Sheynis, Rebecca Thompson, Kevin W. Tipping, Wei-Feng Xue, Neil A. Ranson, Paul A. Beales, Eric W. Hewitt, Sheena E. Radford

**Affiliations:** 1 Astbury Centre for Structural Molecular Biology and School of Molecular and Cellular Biology, University of Leeds, Leeds, United Kingdom; 2 Astbury Centre for Structural Molecular Biology and School of Chemistry, University of Leeds, Leeds, United Kingdom; University of Pittsburgh School of Medicine, United States of America

## Abstract

Although the molecular mechanisms underlying the pathology of amyloidoses are not well understood, the interaction between amyloid proteins and cell membranes is thought to play a role in several amyloid diseases. Amyloid fibrils of β_2_-microglobulin (β_2_m), associated with dialysis-related amyloidosis (DRA), have been shown to cause disruption of anionic lipid bilayers *in vitro*. However, the effect of lipid composition and the chemical environment in which β_2_m-lipid interactions occur have not been investigated previously. Here we examine membrane damage resulting from the interaction of β_2_m monomers and fibrils with lipid bilayers. Using dye release, tryptophan fluorescence quenching and fluorescence confocal microscopy assays we investigate the effect of anionic lipid composition and pH on the susceptibility of liposomes to fibril-induced membrane damage. We show that β_2_m fibril-induced membrane disruption is modulated by anionic lipid composition and is enhanced by acidic pH. Most strikingly, the greatest degree of membrane disruption is observed for liposomes containing bis(monoacylglycero)phosphate (BMP) at acidic pH, conditions likely to reflect those encountered in the endocytic pathway. The results suggest that the interaction between β_2_m fibrils and membranes of endosomal origin may play a role in the molecular mechanism of β_2_m amyloid-associated osteoarticular tissue destruction in DRA.

## Introduction

The aggregation of proteins into amyloid fibrils is associated with many debilitating disorders, including type II diabetes mellitus, Alzheimer’s, Parkinson’s, Creutzfeldt-Jakob disease and dialysis-related amyloidosis (DRA) [1]. The assembly of normally soluble proteins and peptides into amyloid fibrils occurs through a process of nucleated polymerization and elongation [2]–[4] where, irrespective of primary sequence, a common cross-β molecular architecture is adopted [5], [6]. However, fibrils of different morphologies or super-structural features may be formed, even from the same starting material, resulting in an enormous complexity and heterogeneity of species populated during amyloid fibril formation [1], [5], [7], [8].

Numerous studies have linked the cell death and tissue damage associated with amyloid diseases to the existence of oligomers formed early in the process of protein aggregation, rather than late stage amyloid fibrils or plaques (see for example [9]–[11]). However, there is also evidence that fibrils can exhibit cytotoxic potential that is modulated by fibril morphology, fibril length and particle concentration [1], [12]–[15]. Although the molecular and cellular mechanisms of amyloid cytotoxicity remain unclear [1], [16], [17], it is becoming increasingly apparent that cellular membranes are a target for amyloid cytotoxicity [16], [18]. Cellular interfaces, particularly charged membranes, have been shown to promote protein misfolding and fibril formation (see for example [19]–[22]), and numerous studies have indicated that cellular membranes can be susceptible to damage by amyloid species (see for example [23]–[26]).

The specialized lipid compositions of cellular and intracellular membranes facilitate specific exchange of biological materials and enable homeostasis of the internal chemical environment [27]. It has been suggested that amyloid-induced membrane damage may result in disruption of cellular compartmentalization, loss of chemical potential gradients across the membrane, disruption of membrane-mediated signalling pathways and/or energetic dysfunction, leading to amyloid-mediated cytotoxicity (reviewed in [26], [28]). Hence, the lipid composition and charge state of the membrane, and the chemical environment in which amyloid-lipid interactions occur *in vivo*, may play a role in the manifestation of amyloid cytotoxicity.

Here we utilize β_2_-microglobulin (β_2_m), a 99-residue protein with an immunoglobulin fold [29], to investigate the effect of lipid composition and pH on the interaction between amyloid fibrils and lipid bilayers. β_2_m, the light chain of the human major histocompatibility class I complex, forms amyloid fibrils associated with DRA, a debilitating osetoarticular complication of long-term hemodialysis [5], [30]. A single point mutation in the sequence of β_2_m (Asp76Asn) has also been implicated in a hereditary systemic amyloidosis [31]. β_2_m fibrils with a long straight morphology, similar to those observed in *ex vivo* DRA plaques, are readily formed *in vitro* at low pH and low ionic strength [32], [33]. By contrast, at neutral pH the native monomer of β_2_m is unable to assemble into amyloid fibrils (within an experimentally accessible timescale) in the absence of additional additives such as Cu^2+^ [34], heparin [35], trifluoroethanol [36] or lysophospholipids [37].

Upon interaction with asolectin lipid bilayers, monomeric β_2_m has been shown to form relatively non-selective, voltage independent ion channels [38]. Xue et al [14] have also demonstrated that interaction of liposomes with β_2_m fibrils results in membrane damage that is detectable by dye release experiments. This β_2_m fibril-induced membrane damage is also paralleled by a decrease in apparent cell viability, as measured by the reduction of 3-(4,5-dimethylthiazol-2-yl)-2,5-diphenyltetrazolium bromide (MTT) [14]. Mechanical agitation of long straight β_2_m fibrils results in fibril fragmentation, hence an increase in fibril particle concentration and a reduction in average fibril length [39], [40]. At equal monomer-equivalent concentrations, fragmented β_2_m fibrils have been shown to cause greater membrane damage than their unfragmented counterparts, despite the fragmented fibrils maintaining an identical structure [14]. Cryo-electron tomography imaging of fragmented β_2_m amyloid fibrils in the presence of synthetic liposomes has shown that fibril-lipid interactions occur primarily at the fibril ends, resulting in membrane distortion and removal, or blebbing, of the outer membrane leaflet [41]. Membrane damage induced by β_2_m fibrils is also affected by some, but not all, polyphenols and long-chain, but not short-chain, glycosaminoglycans (GAGs) [42], further demonstrating a complex interplay between β_2_m amyloid structure, fibril stability and interactions with lipid membranes.

**Figure 1:**
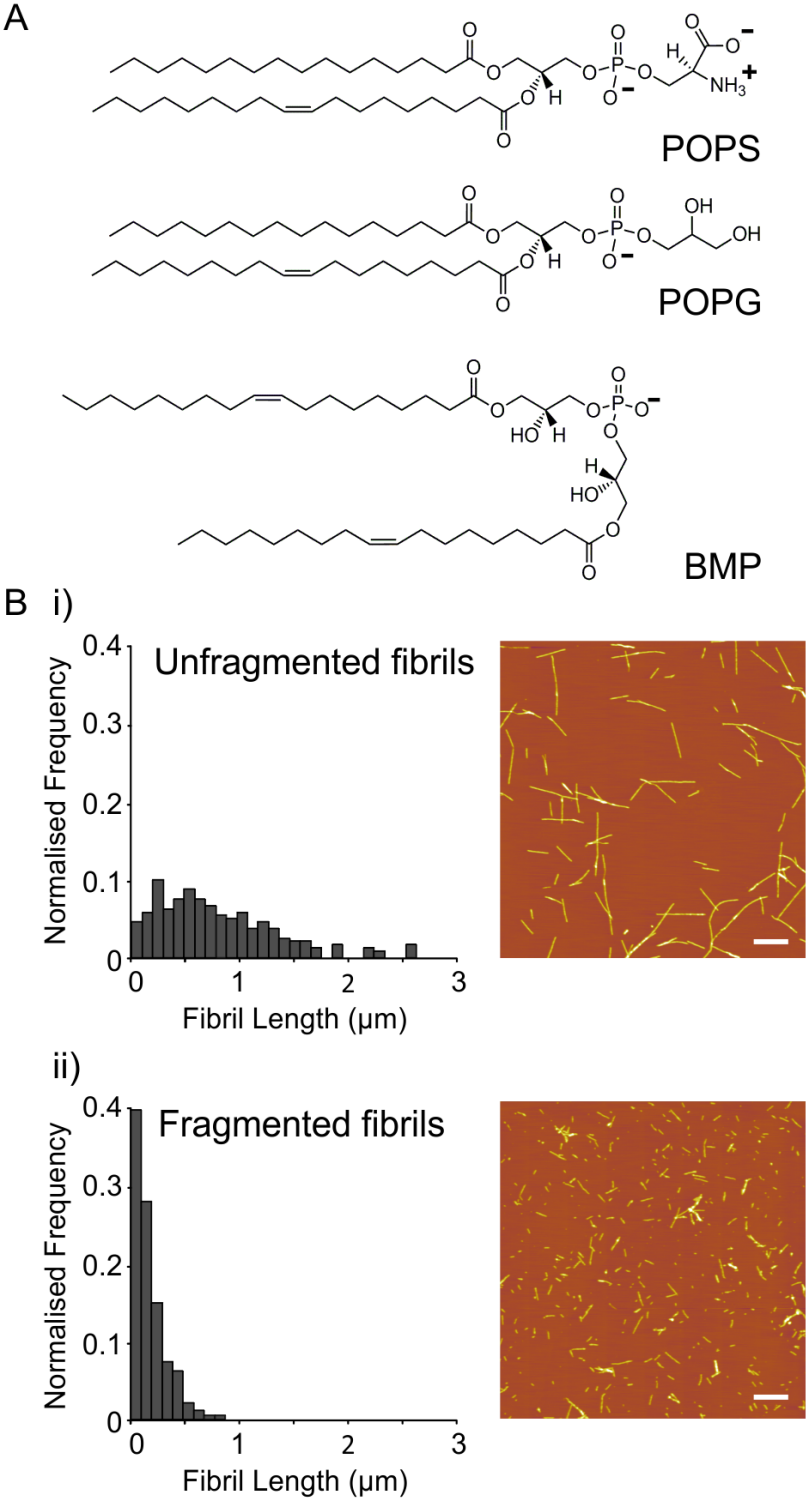
Lipid structures and characterization of β_2_m fibrils using AFM. (*A*) Structure of anionic lipids, POPS, POPG and BMP. (*B*) Fibril length distributions and representative AFM images of (*i*) unfragmented (1.30±0.05 µm) and (*ii*) fragmented (0.30±0.01 µm) β_2_m fibrils. Scale bar 1 µm.

To unravel the mechanism(s) of β_2_m fibril-induced membrane damage, a greater understanding of amyloid-lipid interactions is required. Here we examine membrane damage resulting from the interaction of β_2_m monomers, fragmented and unfragmented fibrils with lipid bilayers using dye release, tryptophan fluorescence quenching and confocal microscopy assays. Liposomes containing the anionic lipids 1-palmitoyl-2-oleoyl-*sn*-glycero-3-phospho-L-serine (POPS), 1-palmitoyl-2-oleoyl-*sn*-glycero-3-phospho-(1′-*rac*-glycerol) (POPG) and bis(monoacylglycero)phosphate (BMP), a structurally unusual lipid also known as lysobisphosphatidic acid which is principally localized to membranes of endosomal origin [43]–[45], were investigated (Fig. 1*A*). We demonstrate that β_2_m fibril-induced membrane damage is modulated by lipid composition and pH, with membrane damage being enhanced by the presence of anionic lipids at acidic pH. Strikingly, compared with POPS-, and to a lesser extent POPG-containing membranes, which are relatively resistant to treatment with β_2_m fibrils, considerable membrane damage is observed for BMP-containing membranes at acidic pH, conditions likely to be encountered by β_2_m amyloid fibrils in the endocytic pathway [45]. Combined with previous experiments which have shown endocytic uptake of β_2_m fibrils by macrophages [46] and the inability of monocytes and macrophages to degrade β_2_m fibrils [46]–[48], the biophysical observations presented here suggest that disruption of endosome function, as a result of β_2_m fibril-lipid interactions, may play a role in β_2_m amyloid pathology.

## Materials and Methods

### Expression and purification of β_2_m

β_2_m was expressed recombinantly in *E. coli* [33] and purified from inclusion bodies as previously described [49]. Purified monomeric β_2_m was dialyzed into deionized water and stored as a lyophilized powder at −20°C. The native monomer of β_2_m has been shown to be stably folded at pH 7.4 [49], [50]. β_2_m monomer was resuspended to a concentration of 120 µM in 10 mM sodium phosphate buffer, 50 mM NaCl, pH 7.4, prepared as fibrils, or fluorescently labeled as described below.

### β_2_m fibril preparation

Long straight β_2_m fibrils were prepared at pH 2.0 by seeding monomers with *de novo* formed fibrils as previously described [14]. Briefly, lyophilized β_2_m monomer was dissolved and diluted to a final concentration of 120 µM in 10 mM sodium dihydrogen phosphate, 50 mM NaCl adjusted to pH 2.0 using HCl. The reaction mixture was immediately syringe-filtered (0.2 µm Minisart fast flow, Sartorious Stedim Biotech) prior to setting up fibril growth reactions. *De novo* formed β_2_m fibrils used for seeding were formed by vigorously agitating the sample for 3 days and unfragmented fibrils were then formed by addition of 0.1% (w/w) seed to 120 µM β_2_m monomer and incubation under quiescent conditions for 48 h. These unfragmented fibrils were subsequently agitated vigorously for 48 h to form fragmented fibrils. All fibrils were prepared at 25°C and all agitation steps were performed by stirring 500 µl of sample in a 1.5 ml glass vial containing a 3×8 mm polytetrafluoroethylene-coated magnetic stirring bar at 1,000 rpm using a custom-made precision stirrer (built by the workshop of the School of Physics and Astronomy, University of Leeds) [14]. Under the conditions employed all fibril solutions were translucent without visible turbidity, indicating that the samples contain well-dispersed fibrils.

### Characterization of fibrils by atomic force microscopy (AFM)

Unfragmented and fragmented β_2_m fibrils were imaged by tapping mode AFM and these images were processed to determine the weight average fibril length and fibril particle concentration using the method described previously [14], [39], [40].

### Preparation of lipid vesicles

Lipids were purchased from Avanti Polar Lipids (Birmingham, Al, USA) as follows: synthetic phospholipids 1-palmitoyl-2-oleoyl-*sn*-glycero-3-phosphocholine (POPC - 850457P), 1-palmitoyl-2-oleoyl-*sn*-glycero-3-phosphoethanolamine (POPE – 850757P), 1-palmitoyl-2-oleoyl-*sn*-glycero-3-phospho-L-serine (POPS – 840034P), 1-palmitoyl-2-oleoyl-*sn*-glycero-3-phospho-(1′-*rac*-glycerol) (POPG – 840457P), bis(monoaclglycero)phosphate (BMP -857133P), 1,2-dioleoyl-*sn-*glycero-3-phospho-L-serine (DOPS – 840035P), 1,2-dimyristoyl-*sn*-glycero-3-phosphoethanolamine-N-(lissamine rhodamine B sulfonyl) (rhodamine labelled DOPE - 810157P), sphingomyelin purified from porcine brain (SM – 860062P); and cholesterol purified from ovine wool (700000P). 1,2-dioleoyl-*sn*-glycero-3-phosphocholine (DOPC – P6354) and 1,2-dioleoyl-*sn*-glycero-3-phospho-(1′-*rac*-glycerol) (DOPG – P9664) were purchased from Sigma-Aldrich (Dorset, UK).

Large unilamellar vesicles (LUVs) were prepared from two different sets of lipid compositions: i) simple lipid mixtures containing 75 mol % POPC or POPG, plus 25 mol % cholesterol; or ii) complex lipid mixtures containing 0, 12 or 50 mol % anionic lipid (POPG, POPS or BMP (Fig. 1*A*)). To enable direct comparison across the different anionic lipid components, the remaining lipids of the complex lipid mixtures were made up of zwitterionic components in a mol/mol ratio of 36 POPC: 20 POPE: 7 SM: 25 cholesterol (Table S1,). Typically, 0.2% mol/mol rhodamine labelled DOPE was also included to enable determination of the lipid concentration. All lipid components were dissolved in chloroform and mixed in the appropriate ratio. The solvent was evaporated under a stream of N_2_ gas to form a thin lipid film over the bottom of a glass tube and the lipid mixture was further dried under vacuum for approximately 3 h. Lipids were rehydrated to a final concentration of 10–25 mM for ≥30 min, typically in *Assay Buffer* (50 mM composite citric acid – monosodium phosphate buffer (Table S2), 107 mM NaCl, 1 mM EDTA) at pH 7.4 unless specified otherwise. The buffer was carefully chosen so as to be iso-osmotically balanced to the CF buffer in the vesicles’ interior (see below), whilst ensuring that the ionic strength did not vary widely over the pH range used. The resulting lipid suspension was typically put through 5 rapid freeze-thaw cycles before being extruded through 400 nm polycarbonate filters using a Mini-Extruder apparatus (Avanti) to produce LUVs. To ensure lipid integrity, all LUVs were stored on ice and used within two days of extrusion.

Giant unilamellar vesicles (GUVs) were prepared by electroformation. Briefly, solutions of 100 mol % DOPC or 80 mol % DOPC plus 20 mol % BMP were prepared in chloroform to a total lipid concentration of 1 mM, supplemented with the lipophilic fluorescent probe 1,1′-dioctadecyl-3,3,3′,3′-tetramethylindodicarbocyanine perchlorate (DiD) (Molecular Probes, D-307) at 0.5 % mol/mol. Yields of GUVs prepared by electroformation using >20 mol % BMP or pH<6.5 were low, and hence these vesicles were unsuitable for confocal microscopy. Aliquots of 70 µl lipid solution were placed dropwise on the platinum wires of an electroformation chamber (built in-house) and dried under vacuum for at least 2 h. The resulting lipid films were hydrated in un-buffered 350 mM sucrose solution. The sucrose concentration inside the vesicles was selected to match the osmolarity of the *Assay Buffer*. The low ionic strength solutions used for GUV preparation are required to ensure optimal vesicle yield. GUVs were formed at room temperature by applying a 2.5 a.c. electric field across the wires at 10 Hz for 45 min, followed by 3 Hz for 20 min, 1 Hz for 7–10 min and 0.5 Hz for 5–7 min. GUVs were then collected from the chamber, stored at 4°C and used within one day of preparation.

### Spectrofluorometric assays

All steady-state florescence emission measurements were performed at 37°C using a QuantaMaster spectrofluorometer (Photon Technology International, West Sussex, UK). Excitation and emission bandwidths were set at 4–8 nm. Tryptophan fluorescence was excited at 290 nm, and fluorescence emission was monitored from 300–500 nm, while the excitation wavelength for carboxyfluorescein (CF) was set to 492 nm and emission was monitored from 500–625 nm or 513 nm for continuous kinetic experiments.

### Carboxyfluorescein dye release assays

The release of CF dye from LUVs was used to measure membrane disruption upon interaction with β_2_m using an adaptation of the method previously described [14]. CF fluorescence is pH sensitive. CF also has limited solubility, particularly at acidic pH. For all dye release experiments presented herein, CF was encapsulated in LUVs at pH 7.4 and high concentration (50 mM), conditions in which CF fluorescence is primarily self-quenched [51]. Lipids were extruding in the presence of 50 mM 5(6)-carboxyfluorescein (21877, Sigma-Aldrich) and excess CF was removed from the exterior of the resulting vesicles by centrifuging several times (20 min, ∼13,000×*g* in a micro-centrifuge) and resuspending the CF-loaded LUV pellet in CF-free buffer, as described below. For each dye release measurement, β_2_m monomers, fragmented or unfragmented fibrils were diluted directly from the original 120 µM stock and incubated with the CF-loaded LUVs in a total volume of 200 µl at pH 4.5–7.4, 37°C. Unless indicated otherwise, a fibril concentration of 6 µM monomer equivalent and LUV concentration of 5 µM lipid molecule equivalent were used. For all experiments, irrespective of the pH of incubation, the pH was adjusted to 7.4 immediately before measuring CF fluorescence. Hence, CF fluorescence was always measured at pH 7.4, allowing direct comparison of the fluorescence arising from CF encapsulated in, and released from, the LUV interior.

CF release kinetics were monitored continuously at pH 7.4 for liposomes consisting of the simple lipid mixtures loaded with *CF Buffer* (50 mM HEPES, 10 mM NaCl, 1 mM EDTA pH 7.4 plus 50 mM CF) on the vesicle interior and *Buffer A* (50 mM HEPES, 107 mM NaCl, 1 mM EDTA pH 7.4) on the vesicle exterior. At pH 4.5, CF release was monitored discontinuously. LUVs were loaded with *CF Buffer* on the vesicle interior and *Buffer B* (50 mM MES, 107 mM NaCl, 1 mM EDTA pH 4.5) was used for the vesicle exterior. A separate sample was measured for each time point. The pH was adjusted by adding 1 ml of *Buffer A* immediately before measuring CF fluorescence.

CF release from the complex lipid mixtures was measured for LUVs loaded with 50 mM sodium phosphate, 10 mM NaCl, 1 mM EDTA pH 7.4 plus 50 mM CF on the vesicle interior, with *Assay Buffer* at pH 4.5, 5.5, 6.5 or 7.4 (Table S2) on the vesicle exterior. Following 10 min incubation with the protein, the pH of each CF release sample was adjusted by adding 1 ml *Assay Buffer* at pH 7.4 immediately before measuring CF fluorescence.

For all CF release measurements, the percentage of dye release was determined as the ratio of fluorescence intensity upon dye release (*F*) to the CF fluorescence of maximal dye release (*F_T_*) measured after dissolution of the lipid vesicles with 2% (v/v) Triton X-100 incubated at 37°C for >3 min. All CF fluorescence emission values were taken at 513 nm, normalized based upon *F_T_*, and corrected to account for background emission arising from CF in the LUV interior, any inherent vesicles ‘leakiness’ and inner filter effects from vesicle scattering using an equivalent CF-loaded LUV only blank sample (*F_B_*) as follows:
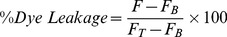
(1)


For all experiments, *F_B_* was <15% of *F_T_*.

### Tryptophan fluorescence quenching

Quenching of intrinsic tryptophan fluorescence of β_2_m samples in the presence of acrylamide was used to probe the interaction between β_2_m monomers, fragmented or unfragmented fibrils and lipid membranes at pH 4.5 and pH 7.4. For each acrylamide concentration, two samples containing β_2_m (6 µM monomer equivalent concentration) were prepared by dilution into *Assay Buffer* from the original 120 µM stock; i) in the absence of LUVs and ii) in the presence of LUVs (5 µM lipid molecule concentration) comprising 0, 12 or 50 mol % BMP plus the complex zwitterionic lipid mixture. Following incubation at 37°C for 10 min, ultrapure acrylamide (01696, Sigma-Aldrich) was titrated from an aqueous 5 M stock to give an acrylamide concentration between 10 and 250 mM ([*Q*]). Tryptophan fluorescence was then measured immediately.

The efficiency of quenching (*F_0_/F*), for each sample at [*Q*], was calculated by dividing the tryptophan fluorescence intensity at 340 nm in the absence of quencher (*F_0_*) by the fluorescence emission at 340 nm in the presence of the acrylamide quenching agent (*F*). Linear regression was performed using the Stern–Volmer equation for a dynamic process to determine the *K_SV_* for each sample, as follows:
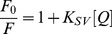
(2)


Δ*K_SV_* was calculated by subtracting *K_SV_* obtained for β_2_m monomers, fragmented or unfragmented fibrils in the absence of LUVs from the corresponding *K_SV_* values obtained for LUV-containing samples.

### Preparation of fluorescently labeled β_2_m

To enable confocal imaging, β_2_m monomer was labeled with the fluorescent dye tetramethylrhodamine (TMR) as follows. 5-(and-6)-carboxytetramethylrhodamine, succinimidyl ester (C1171, Molecular Probes) was freshly dissolved in DMSO at 1 mg/ml and a ten-fold molar excess was added (dropwise whilst stirring) to β_2_m monomer resuspended in 10 mM sodium bicarbonate, pH 9.4. The labeling reaction was allowed to proceed in the dark for 1 h at ambient temperature and was stopped by adding a five-fold molar excess of Tris-HCl pH 8.0 over the concentration of the dye. Unbound dye was separated from the β_2_m-TMR conjugate using a PD10 desalting column (GE Healthcare, Little Chalfont, UK) in 25 mM sodium phosphate buffer, pH 7.5. β_2_m was labeled with 2, 3, 4 or 5 TMR molecules per monomer with a ratio of ∼1∶2∶2∶1 as determined by electrospray ionization mass spectrometry (ESI-MS). The TMR-labeled β_2_m was concentrated to 0.7 mM, snap frozen in liquid nitrogen and stored at −80°C. For confocal experiments, 10 mol % TMR-labeled β_2_m monomer was mixed with 90 mol % unlabelled monomer and fibrils were subsequently formed as described above.

### Fluorescence confocal microscopy

Interactions between β_2_m and GUVs were visualized using fluorescence confocal microscopy. DiD-labeled GUVs were diluted five-fold in *Assay Buffer* (pH 6.5 or 7.4) and 100 µl of the suspensions were supplemented with 10 µM CF in *Assay buffer* pH 7.4 and mixed with either 1.0 µl of fibril growth buffer (10 mM sodium dihydrogen phosphate, 50 mm NaCl, pH 2.0) for control experiments, 1.0 µl of TMR-labeled β_2_m fragmented fibrils (1.2 µM monomer equivalent) or 10 µl of TMR-β_2_m monomers (11 µM). The monomers were assayed at a higher concentration than fibrils to enable protein visualization under the confocal microscope. Total lipid concentration was 3.3 µM. The samples were incubated for 15 min on a glass-bottom culture dish (MatTek Corp, P35G-1.5-20-C) at ambient temperature and imaged on Zeiss LSM 700 confocal microscope using a Zeiss 63x/1.4 N.A. DIC Plan Apochrom oil immersion lens. Culture dishes were pre-treated with 10% (w/w) bovine serum albumin solution (Sigma-Aldrich) to prevent adsorption of lipids to the glass. CF, DiD and TMR probes were excited by lasers at 488 nm, 555 nm and 639 nm, respectively.

### Dynamic light scattering

Size distribution measurements of extruded vesicles were performed using dynamic light scattering (DLS or Quasi-Elastic-Light-Scattering - QELS) on a miniDAWN TREOS system, equipped with a Wyatt QELS detector (Wyatt Technology), run in batch mode at room temperature. Lipids were prepared and extruded in *Assay Buffer* pH 7.4 before being subjected to the same washing procedure used to remove CF from the vesicle exterior, as described in *Carboxyfluorescein dye release assays*, above. DLS was measured for LUVs resuspended at ∼50 µM lipid concentration in *Assay Buffer* at either pH 4.5 or 7.4. For each sample, three measurements were performed, collecting QELS data at 5 s intervals for 3 min. Data were processed by regularization analysis using Wyatt ASTRA 6.0 software. Hydrodynamic radii (*R_h_*) obtained from DLS data are reported as the weighted average of three experiments for each sample with an error of 1 S.D.

### Cryogenic transmission electron microscopy

Cryogenic transmission electron microscopy (cryo-EM) was used to visualize extruded LUVs. The LUVs were fixed in vitreous ice on Quantifoil R 3.5, 300 mesh, Cu grids using a Vitrobot mark IV. Cryo-EM was carried out at liquid nitrogen temperatures using a Gatan 626b cryo-holder and a FEI Tecnai-F20 electron microscope. Images were recorded on a Gatan US4000 CCD camera under low-dose conditions at a nominal magnification of 9600 x.

## Results

### Characterization of β_2_m fibrils

Unfragmented fibrils with a weight average length of 1.30±0.05 µm (1 S.E., sample size = 242 fibrils) were formed from recombinantly expressed β_2_m monomer by seeded fibril elongation at pH 2.0 (Fig. 1*B* *(i)*). Fragmented β_2_m fibrils were subsequently formed by vigorous agitation to decrease the weight average length to 0.30±0.01 µm (1 S.E., sample size = 763 fibrils) (Fig. 1*B* *(ii)*).

The protein-to-lipid ratio typically utilized for the dye release experiments described (6 µM monomer equivalent β_2_m: 5 µM lipid molecule equivalent LUVs) is significantly higher than that typically used to monitor membrane damage caused by membrane active peptides, for which a peptide: lipid molar ratio in the region of 1∶10–1000 is typically utilized (see for example [52]). However unlike typical membrane active peptides, β_2_m fibrils are large multimeric aggregates. Assuming the β_2_m fibrils have an average mass/unit length of 53 kDa/nm [53] and based on the fibril length distributions measured herein (Fig. 1*B*), at a β_2_m monomer equivalent concentration of 6 µM the molar fibril particle concentration [40] for fragmented and unfragmented fibrils was determined to be approximately 15.5 nM and 3.4 nM, respectively. Hence the fibril particle: lipid molar ratio used herein equates to ∼1∶300 for fragmented fibrils and ∼1∶1500 for unfragmented fibrils. Assuming each LUV consists of approximately 1.4×10^6^ lipid molecules (400 nm unilamellar vesicles, 5 nm bilayer thickness, 0.7 nm^2^ head-group surface area) this represents an absolute particle ratio of approximately 1000 unfragmented fibrils, 4000 fragmented fibrils or 2×10^6^ β_2_m monomers for every LUV.

### β_2_m fibril-induced dye release is modulated by membrane composition and pH

**Figure 2:**
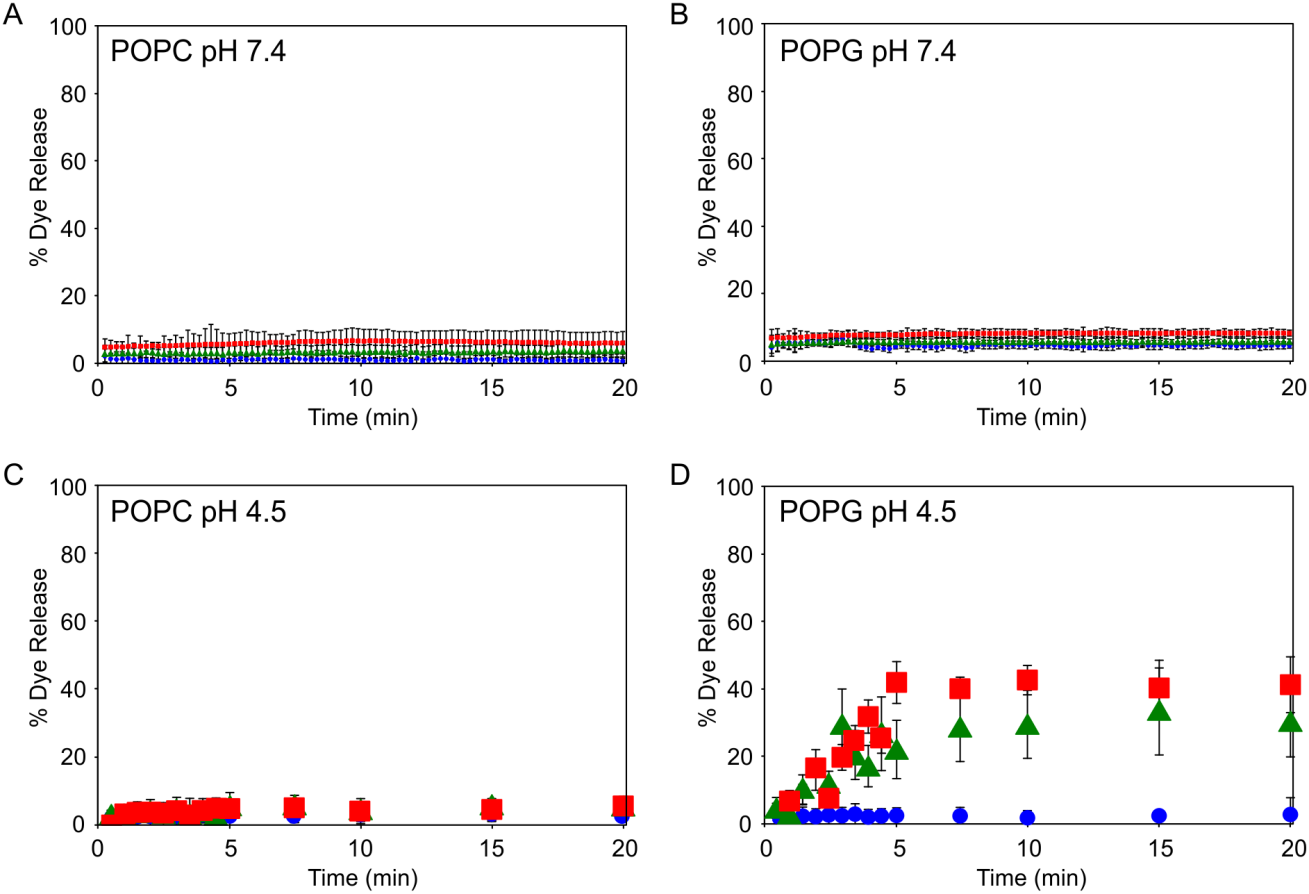
Dye release upon addition of β_2_m to POPC/cholesterol or POPG/cholesterol LUVs. Dye release from LUVs (5 µM lipid) comprising of (*A*) 75 mol % POPC: 25 mol % cholesterol and (*B*) 75 mol % POPG: 25 mol % cholesterol measured continuously over 20 min following addition of 6 µM β_2_m samples in *Buffer A* at pH 7.4, 37°C. Error bars represent 1 standard deviation (S.D.) from three replicates for each β_2_m species. Dye release from LUVs (5 µM lipid) comprising (*C*) 75 mol % POPC: 25 mol % cholesterol and (*D*) 75 mol % POPG: 25 mol % cholesterol measured discontinuously over 20 min following addition of 6 µM β_2_m samples in *Buffer B* at pH 4.5, 37°C. Error bars represent 1 S.D. from three replicates for each β_2_m species at each time point. β_2_m monomer (*circle*, *blue*), fragmented fibrils (*square*, *red*) and unfragmented fibrils (*triangle*, *green*).

To examine the effect of lipid charge and pH on β_2_m fibril-induced membrane damage, dye release from LUVs comprised of 75 mol % POPC plus 25 mol % cholesterol (zwitterionic) and 75 mol % POPG plus 25 mol % cholesterol (anionic) was measured. Upon addition of β_2_m monomers, fragmented or unfragmented fibrils, CF dye release was monitored at pH 7.4, either after continuous incubation at pH 7.4 (Fig. 2*A* and *B*) or in a discontinuous manner after incubation at pH 4.5 and subsequent immediate dilution to adjust the pH to 7.4 before measuring dye release for individual samples at each time point (Fig. 2*C* and *D*, see *Materials and Methods*). For all lipid mixtures and pH values investigated, dye release resulting from the addition of monomers is minimal (<10%), despite the high protein: lipid ratio used, consistent with previous results [14]. Upon addition of β_2_m fibrils, <10% dye release is observed from LUVs of the POPC lipid mixture regardless of pH (Fig. 2*A* and *C*). Similarly, fibril-induced dye release from LUVs of the POPG lipid mixture is minimal at pH 7.4 (Fig. 2*B*). Considerable dye release is only observed upon addition of fragmented fibrils (up to ∼40% dye release) or unfragmented fibrils (up to ∼30% dye release) to POPG-containing LUVs at pH 4.5 (Fig. 2*D*). Dye release is typically observed to plateau approximately 10 min after addition of the β_2_m fibrils and no further increase in dye release is observed after 2 h (Fig. S1). Accordingly, all subsequent dye release measurements were taken 10 min after the addition of protein to the LUVs. Although ∼5% β_2_m monomer persists following fibril maturation [54], previous experiments have shown that fibrils formed as described herein lack oligomeric species (detectable by size exclusion chromatography or recognized by the A11 antibody [14]). Hence, it is unlikely that residual oligomers from the fibril preparation are the culprits of the membrane disruption effects observed. Instead, membrane disruption is caused by the presence of β_2_m fibrils, either by direct interaction of the LUVs with the fibrils themselves, or through species formed upon incubation of the fibrils with the liposomes [14].

**Figure 3:**
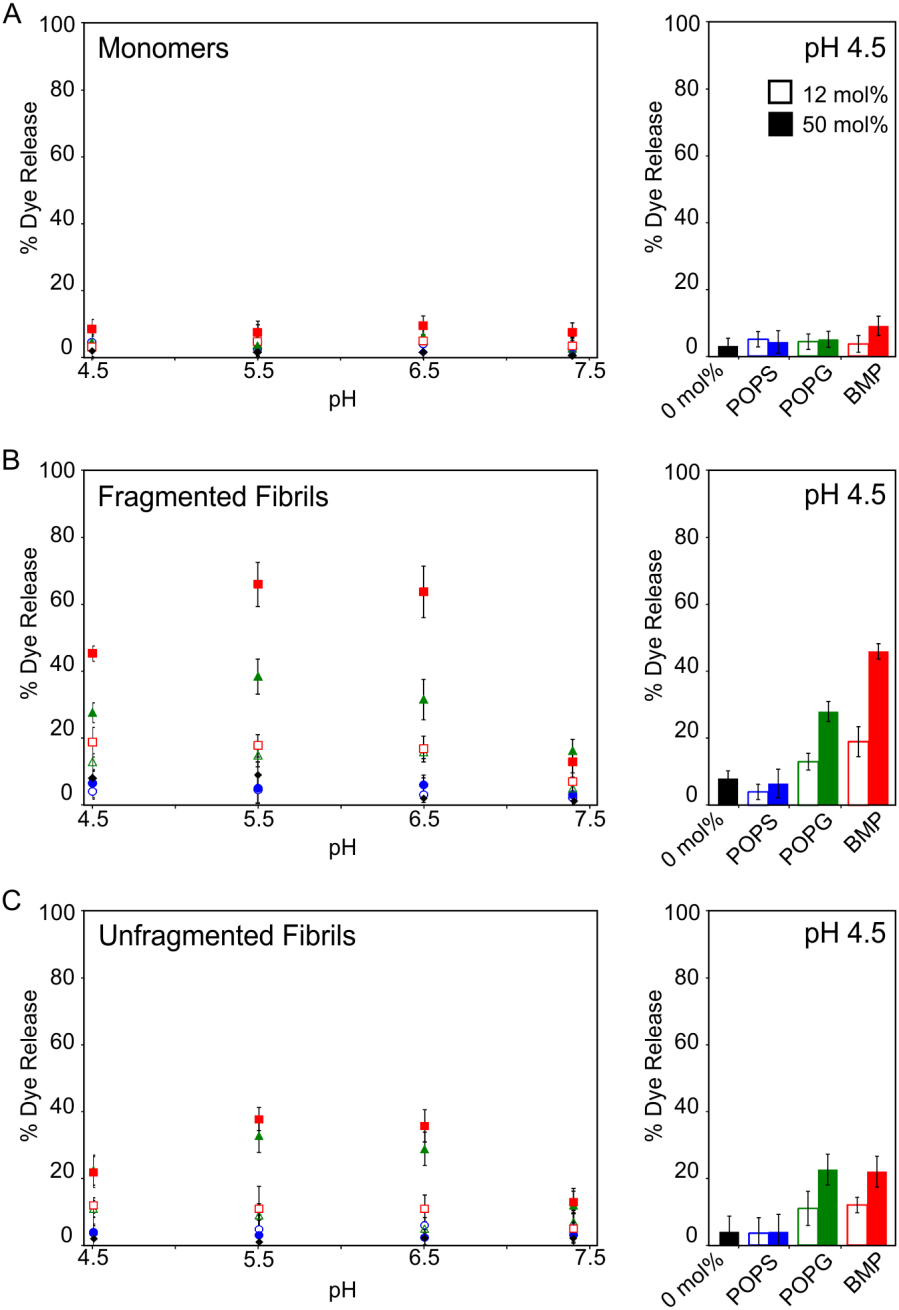
Dye release from LUVs comprised of complex anionic lipid mixtures upon addition of β_2_m samples at pH 4.5–7.4. Dye release was measured 10 min after the addition of 6 µM (monomer equivalent concentration) of (*A*) β_2_m monomers, (*B*) fragmented fibrils or (*C*) unfragmented fibrils added to CF-loaded LUVs (5 µM lipid) in *Assay Buffer* pH 4.5–7.4, 37°C. LUVs are comprised of 36 POPC: 20 POPE: 7 SM: 25 cholesterol (mol/mol) doped with 0 mol % (*diamond, black*), 12 mol % (*open symbols*) or 50 mol % (*solid symbols*) anionic lipid, POPS (*circle, blue*), POPG (*diamond, green*) or BMP (*square, red*). Corresponding bar graph of dye release at pH 4.5 in 0 mol % (*black*), 12 mol % (*open bar*) or 50 mol % (*solid bar*) POPG, POPS or BMP are shown alongside. Error bars represent 1 standard error (S.E.) from three independent repeats, each of three replicates.

The data shown in Fig. 2 suggest that the extent of membrane damage conferred by β_2_m fibrils is modulated by lipid charge and pH. Hence, a systematic study of different, more physiologically relevant, complex anionic lipid mixtures was performed over a physiologically relevant pH range. The % dye release from LUVs comprised of a complex zwitterionic lipid mixture, supplemented with increasing concentrations of POPS, POPG, or BMP was measured at pH 4.5–7.4 for β_2_m monomers, fragmented or unfragmented fibrils as shown in Fig. 3*A*–*C*, respectively. The complex lipid mixtures utilized here were designed to enable direct comparison of the different anionic lipids, whilst also reflecting an approximate global average of the zwitterionic lipid components present in cellular membranes enriched in POPS (plasma membrane), POPG (mitochondrial membranes) and BMP (endocytic membranes) [27].

Minimal membrane damage (<10% dye release) is observed upon the addition of β_2_m monomers to LUVs regardless of lipid composition and pH (Fig. 3*A* *,* Table S3). Likewise, for both fragmented and unfragmented fibrils, minimal dye release occurs with the zwitterionic control lipid mixture (i.e. 0 mol % anionic lipid, *shown in black*) or POPS-containing LUVs (*shown in blue*, Fig. 3*B* and *C*). However, LUVs containing BMP (*shown in red*), and to a lesser extent POPG-containing LUVs (*shown in green*), are susceptible to membrane disruption by β_2_m fibrils (Fig. 3*B* and *C*, Table S3).

The extent of dye release from POPG- and BMP-containing LUVs increases significantly as the anionic lipid concentration is increased from 12 mol % to 50 mol % (Fig. 3*B* and *C* *, open* and *closed symbols*, respectively). For fragmented fibrils at pH 4.5 (see Fig. 3*B*, *bar graph*), the greatest % dye release is observed for LUVs containing BMP (46±2% for 50 mol % BMP and 19±5% for 12 mol % BMP), whereas the % dye release observed for POPG-containing membranes is typically lower than the equivalent BMP-containing LUVs (28±3% for 50 mol % POPG and 13±3% for 12 mol % POPG). A similar trend in % dye release is observed for unfragmented fibrils at pH 4.5 however, in this case, the % dye release measured is typically lower than that observed for fragmented fibrils (Fig. 3*B*, *bar graph*) (22±5% for 50 mol % BMP, 12±2% for 12 mol % BMP, 23±5% for 50 mol % POPG and 11±5% for 12 mol % POPG).

The extent of membrane disruption also varies as a function of pH. For all lipid compositions studied dye release is minimal at pH 7.4 for both fragmented and unfragmented fibrils. Maximal dye release from POPG- and BMP-containing LUVs is observed for β_2_m fibrils at pH 5.5–6.5, with the extent of dye release observed at pH 4.5 typically being lower.

**Figure 4:**
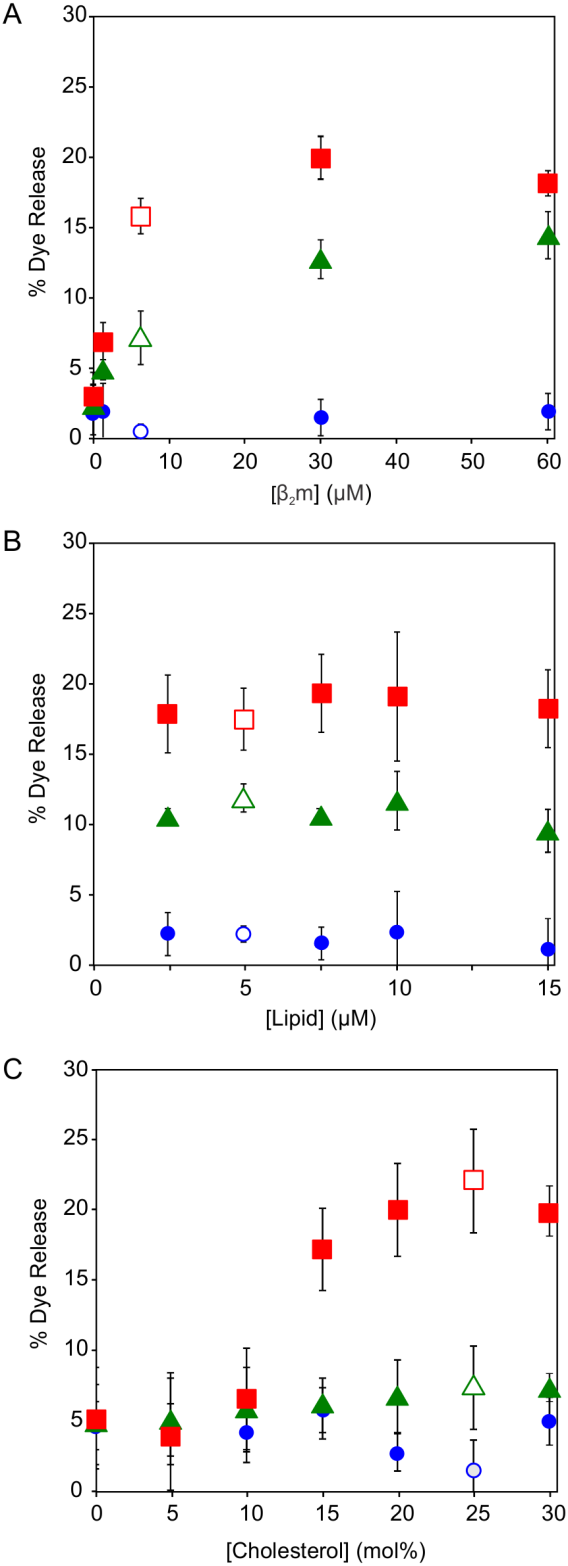
Dye release from LUVs comprised of 12 mol % BMP at pH 4.5, varying β_2_m: lipid concentration ratio and cholesterol content. (*A*) Dye release measured for LUVs (5 µM lipid) at different monomer equivalent concentrations of β_2_m. (*B*) Dye release measured for varying lipid equivalent concentrations of LUVs at 6 µM monomer equivalent concentration of β_2_m. (*C*) Dye release from LUVs (5 µM lipid) comprising 36 POPC: 20 POPE: 7 SM (mol/mol), 12 mol % BMP plus varying concentrations of cholesterol at 6 µM monomer equivalent concentration of β_2_m. Dye release was measured 10 min after addition β_2_m monomer (*circle, blue*), fragmented fibrils (*square, red*) and unfragmented fibrils (*triangle, green*) in *Assay Buffer* pH 4.5, 37°C. Error bars represent 1 S.D. from three replicates. Open symbols corresponds to dye release measured for 6 µM monomer equivalent β_2_m samples with 5 µM lipid equivalent concentration of LUVs containing 25 mol % cholesterol, as in Fig. 3.

The extent of dye release from LUVs comprised of 12 mol % BMP was measured at pH 4.5 for β_2_m concentrations ranging from 0–60 µM monomer equivalent (Fig. 4*A*). For all samples the extent of dye release observed does not increase substantially for protein concentrations ≥6 µM. In addition at a constant 6 µM monomer equivalent β_2_m concentration, the maximum % dye release from LUVs comprised of 12 mol % BMP at pH 4.5 is not altered by varying the concentration of lipids up to 15 µM for all β_2_m species investigated (Fig. 4*B*).

The effect of cholesterol on β_2_m-induced dye release was also measured (Fig. 4*C*). For LUVs comprised of 12 mol % BMP at pH 4.5, ≥10% dye release is only observed for fibrils at ≥15 mol % cholesterol. As a consequence of our approach of varying the anionic lipid concentration whilst maintaining the same molar ratio of zwitterionic components described above, the total molar concentration of cholesterol was varied in different samples (14–28 mol %, see Table S1). As shown in Fig. 4*C*, the presence of 15–30 mol % cholesterol does not alter the extent of dye release observed when either β_2_m monomers or fibrils. Thus, the increase in dye release observed when fibrils are incubated with LUVs containing anionic lipids at acidic pH can be attributed to the presence of BMP, rather than changes in the relative proportion of cholesterol in each sample.

The presence of BMP can alter the size and morphology of extruded LUVs and enable the spontaneously formation of small (<100 nm) vesicles [55], [56]. The size distribution and morphology of extruded LUVs consisting of the POPC/cholesterol and POPG/cholesterol (as employed in Fig. 2) and complex zwitterionic lipid mixture with BMP and cholesterol (as employed in Fig. 3 and 4) were analyzed (Fig. S2 and S3, respectively and Table S4). All vesicles were prepared exactly as described for the dye release experiments and extruded under the same conditions (*Materials and Methods*). No gross differences in morphology were observed by cryo-EM for the different lipid mixtures at either pH 4.5 or 7.4. An average *R_h_* of ∼200 nm was measured by DLS at pH 4.5 or 7.4 for all lipid mixtures (Table S4), consistent with the 400 nm diameter of the pores of the extrusion membrane. The inclusion of different amounts of cholesterol does not affect extruded vesicle size (*R_h_* = ∼160 nm for LUVs containing 12 mol % BMP and either 0 or 25 mol % cholesterol). However, a small decrease in average vesicle *R_h_* was observed with increasing BMP concentration (∼175 nm for 0 mol % BMP compared with ∼125 nm for 50 mol % BMP, averaged across both pH values). To determine whether this decrease in R*_h_* could account for the increase in dye release observed in BMP-containing vesicles, LUVs consisting of the complex zwitterionic lipid mixture plus 12 mol % BMP extruded at 100 nm were also analyzed by DLS and cryo-EM (Fig. S4, Table S4). These LUVs are significantly smaller (*R_h_* = ∼60 nm) than the 400 nm extruded vesicles. However, unlike the BMP-containing LUVs where smaller vesicles (with higher BMP concentration) result in greater dye release, a lower % dye release is observed for the 100 nm extruded LUVs, compared with the equivalent 400 nm LUVs at the same lipid concentration (Fig. S5*A*). Therefore, vesicle size, and hence membrane curvature, is not a defining factor in determining the efficiency of fibril-induced membrane damage in this case.

The biophysical properties of the membrane are also defined by the length and saturation of the acyl chain. Both acyl chains of the 18∶1 form of BMP utilized here are unsaturated, whereas POPS and POPG consist of one saturated and one unsaturated acyl chain. Dye release was also measured for LUVs consisting of 36 POPC: 20 POPE: 7 SM: 25 cholesterol (mol/mol) doped with either 12 mol % DOPS or DOPG. DOPS and DOPG possess the same serine and glycerol headgroups as POPS and POPG, respectively, but both lipid chains are unsaturated, as in BMP (Fig. S5*B* and *C*). Like the equivalent POPS-containing lipid mixture (Fig. 3), no substantial dye release was observed for the DOPS-containing LUVs in the presence of fragmented fibrils. Comparable % dye release was observed for vesicles comprising the POPG (Fig. 3) and DOPG lipid mixtures.

### Dye release is not simply related to the extent of β_2_m-membrane interaction

**Figure 5:**
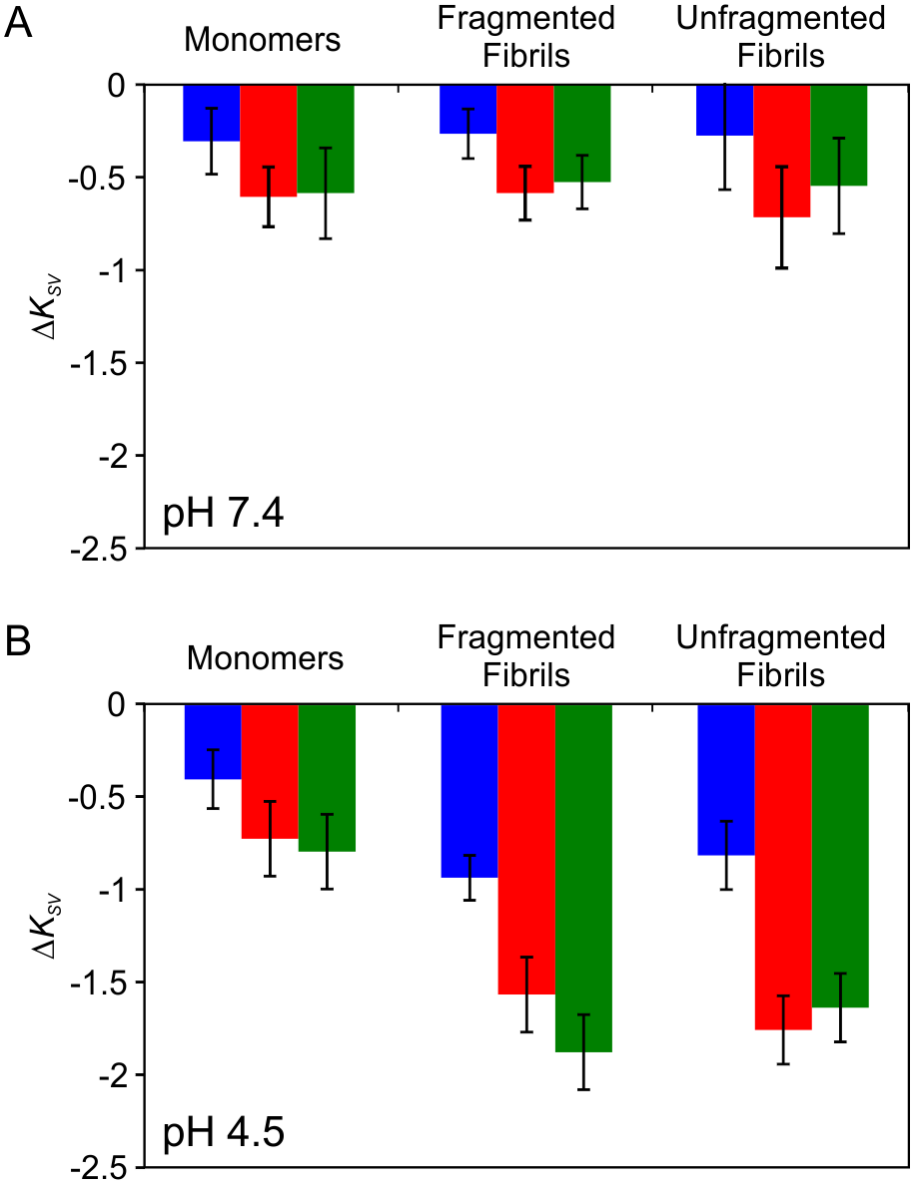
Change in tryptophan fluorescence quenching for β_2_m in the presence of LUVs. Δ *K_SV_* for 6 µM monomer equivalent concentration β_2_m monomer, fragmented or unfragmented fibrils 10 min after addition of LUVs (5 µM lipid) at 37°C in (*A*) *Assay buffer* at pH 7.4 or (*B*) *Assay buffer* at pH 4.5. Lipid mixes comprise 36 POPC: 20 POPE: 7 SM: 25 cholesterol (mol/mol) doped with 0 mol % (*blue*), 12 mol % (*red*) or 50 mol % (*green*) BMP. Error bars represent 1 S.E. from linear regression.

To investigate membrane association of β_2_m monomers, fragmented and unfragmented fibrils, quenching of intrinsic tryptophan fluorescence by acrylamide was utilized. Acrylamide does not readily partition into lipid bilayers. Thus, the Stern-Volmer quenching constant (*K_SV_*) is a reliable reflection of the biomolecular rate constant for dynamic quenching of tryptophan residues accessible to the aqueous phase, where a greater *K_SV_* is observed for more solvent exposed tryptophan residues [57]. Hence, a difference between the *K_SV_* values observed for β_2_m samples in the absence or presence of LUVs is indicative of a change in local environment of Trp-60 and/or Trp-95 in each sample, presumably reflective of an interaction between β_2_m monomers/fibrils and the lipid bilayer. Linear regression of Stern-Volmer plots (Fig. S6 and Table S5,) was performed to determine the change in tryptophan quenching (Δ*K_SV_*) between β_2_m in solution and upon addition of LUVs comprising of 0, 12 or 50 mol % BMP at pH 7.4 or 4.5 (Fig. 5*A* and *B*, respectively).

Only a small decrease in *K_SV_* (Δ*K_SV_* = ∼−0.5 M^−1^) is observed for β_2_m monomers, fragmented or unfragmented fibrils in the presence of the membrane at pH 7.4, regardless of lipid composition. However, at pH 4.5 a larger decrease in *K_SV_* (Δ*K_SV_* = ∼−1.7 M^−1^) is observed for both fragmented and unfragmented β_2_m fibrils in the presence of BMP-containing LUVs; conditions for which significant dye release was also observed. However, by contrast with the results of the dye release experiments, no correlation between Δ*K_SV_* and either fibril length or BMP concentration is observed.

### Visualizing β_2_m fibril-membrane interactions by confocal microscopy

**Figure 6:**
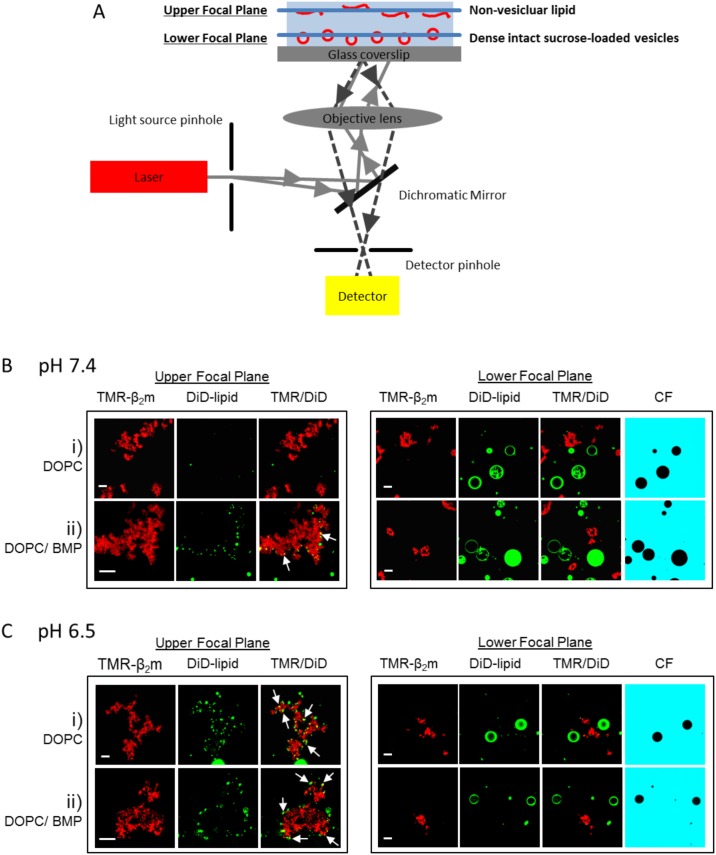
Confocal fluorescence microscopy of GUVs upon addition of fragmented β_2_m fibrils. (*A*) Confocal images were collected in two different focal planes. The upper plane contains mainly non-vesicular lipid while dense, intact sucrose-loaded GUVs are observed mostly in the lower focal plane. Confocal images of TMR-labeled β_2_m fragmented fibrils incubated with DiD-labeled GUVs for 15 min at ambient temperature in (*B*) *Assay Buffer* at pH 7.4 or (*C*) *Assay Buffer* at pH 6.5. *(L*-*R)* TMR-labeled β_2_m fibrils (*red*), DiD-labeled GUVs (*green*), superimposition of the TMR and DiD channels. Soluble CF added to the vesicle exterior is shown in blue *(lower focal plane only*). Representative images when viewed in the upper focal plane and lower focal plane are shown for GUVs comprised of (*i*) 100 mol % DOPC or (ii) 80 mol % DOPC plus 20 mol % BMP. White arrows highlight areas of lipids bound to fibril aggregates. Scale bar 10 µm.

To enable direct visualization of β_2_m-lipid interactions, DiD-labeled GUVs 10–25 µm in diameter were formed from 100 mol % DOPC (zwitterionic) or 80 mol % DOPC plus 20 mol % BMP (anionic) lipid compositions by electroformation (Fig. S7*A*). These GUVs were visualized in the presence of TMR-labeled β_2_m monomers or fragmented fibrils via confocal microscopy in *Assay Buffer* at pH 7.4 and 6.5. Note that GUVs were not able to be prepared at lower pH values or higher concentrations of BMP (*Materials and Methods*). Images were collected in a lower focal plane, which contained mainly dense, intact, sucrose-loaded GUVs, and an upper focal plane, which contained mainly non-vesicular lipid (Fig. 6*A*).

Upon addition of β_2_m monomers, no co-localization of β_2_m with DOPC GUVs or DOPC/BMP GUVs is observed at either pH (Fig. S7*B*–*C*). CF and β_2_m monomers are also excluded from the interior of the GUVs suggesting that membrane damage does not occur upon addition of monomer. Intensity profiles of TMR fluorescence also show that β_2_m monomers do not accumulate on the lipid surface (Fig. S7*B*–*C*).

Upon addition of fragmented β_2_m fibrils to DOPC GUVs at pH 7.4, no co-localization of fibrils and lipids is observed (Fig. 6*B* *(i)*). However, for DOPC vesicles at pH 6.5 (Fig. 6*C* *(i)*) and BMP-containing GUVs at both pH 7.4 and 6.5 (Fig. 6*B* *(ii)* and *C(ii)*), small lipid assemblies (<∼2 µm in size), presumably arising from disruption of GUVs, are observed on the fibril surfaces when viewed in the upper focal plane (*white arrows*). In the lower focal plane, intact GUVs (in which CF is excluded from the vesicle interior) are also observed for both the DOPC- and BMP-containing lipid mixtures regardless of pH (Fig. 6*B* and *C*), indicating the presence of intact GUVs even under conditions in which some membrane damage is present. The co-localization of fibrils with smaller lipid structures suggests that the interaction between β_2_m fibrils and GUVs causes disruption of the GUV structure, consistent with dye release observed for BMP-containing LUVs under similar conditions. However, it is unclear whether this fibril-lipid association arises secondary to, or during, disruption of the vesicle structure. Intriguingly, GUVs in which CF has partially leaked into the vesicle are absent in the microscopy experiments (Fig. 6). This suggests that β_2_m fibril-induced membrane damage results in complete disruption of the membrane architecture, rather than the formation of (meta)stable pores or defects in the lipid bilayer.

The presence of intact GUVs under conditions in which membrane damage has occurred indicates that not all GUVs are susceptible to membrane damage. Indeed, β_2_m fibril-induced dye release from LUVs is also observed to plateau at a maximum of <100 % (Fig. 2) and does not significantly increase after >2 h (Fig. S1). Additionally, the maximum % dye release is not significantly altered by increasing the β_2_m fibril concentration 10-fold or by altering the concentration of LUVs (Fig. 4*A* and *B*). The inability to induce complete dye release at infinite time despite a significant excess of protein, is a perplexing facet of the membrane-damaging mechanism of other aggregated samples (see for example [13], [58]). The mode of membrane damage is likely to be complex due to an equilibrium of membrane bound fibrils and those in the bulk aqueous phase. Fibrils coalesce during incubation (as shown by confocal microscopy in Fig. 6*B* and *C*), resulting in a reduction in exposed fibril surface area. Similarly, upon membrane damage fibrils become coated with lipid (also shown in Fig. 6 *B(ii)* and *C(ii* and *iii)*) which may then reduce or eliminate their ability to induce membrane damage. However, a detailed mechanism of β_2_m fibril-induced membrane damage has not yet been fully elucidated.

## Discussion

### β2m fibril-induced membrane disruption is enhanced by the presence of anionic lipids at acidic pH

The interaction between amyloid fibrils and cellular membranes is thought to be an important facet of several amyloid diseases [16], [18]. Using dye release experiments we have previously shown that β_2_m fibrils cause damage to anionic lipid membranes [14]. However, the effect of lipid composition and the chemical environment in which β_2_m fibril-lipid interactions occur have not been investigated previously.

Here, dye release is observed upon the addition of β_2_m fibrils to synthetic 75 mol % POPG, 25 mol % cholesterol (anionic) LUVs at pH 4.5. However, for 75 mol % POPC, 25 mol % cholesterol (zwitterionic) LUVs no dye release is observed for any β_2_m species at either pH 7.4 or 4.5 (Fig. 2*A* and *C*). Hence, it would appear that the presence of negatively charged lipids is required to render the membrane susceptible to β_2_m fibril-induced damage. Using more complex lipid mixtures containing the anionic lipids POPS, POPG or BMP, we demonstrate that the extent of β_2_m fibril-induced membrane damage is dependent on the identity of the anionic lipid and the pH at which the fibril-lipid interactions occur. Strikingly, fragmented β_2_m fibrils, high BMP concentration, and low pH result in enhanced susceptibility to membrane damage.

**Figure 7:**
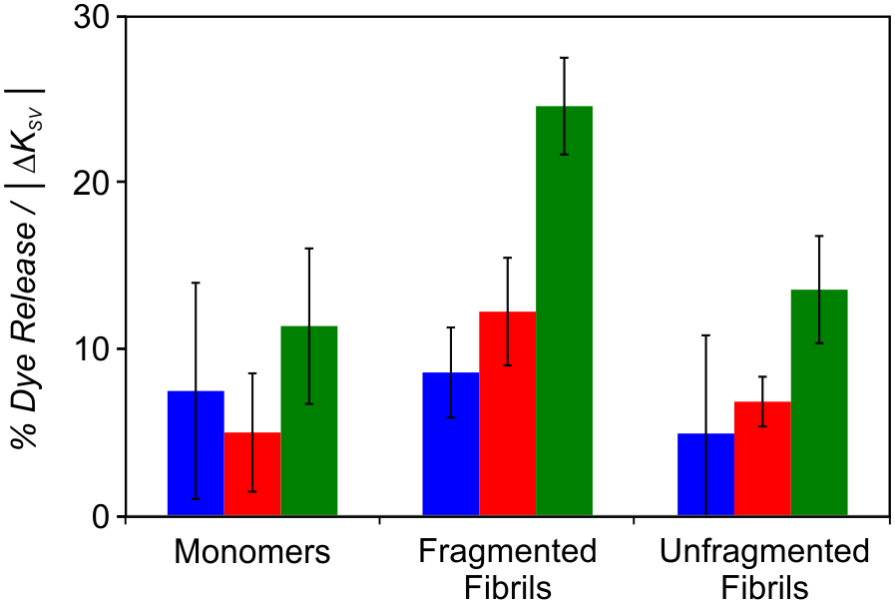
Dye release normalized to the change in tryptophan fluorescence quenching measured for β_2_m with BMP-containing LUVs at pH 4.5. The ratio of % dye release per membrane interaction detected via a change in tryptophan quenching observed in the absence or presence of lipid. β_2_m monomers, fragmented and unfragmented fibrils (6 µM monomer equivalent concentration) were incubated in *Assay Buffer* pH 4.5 with LUVs (5 µM lipid) comprising 36 POPC: 20 POPE: 7 SM: 25 cholesterol (mol/mol) plus 0 mol % (*blue*), 12 mol % (*red*) or 50 mol % (*green*) BMP for 10 min at 37°C. Error bar represent 1 S.E.

In principle, dye release requires that β_2_m interacts directly with the lipid bilayer to give rise a defect in membrane integrity. However, no direct correlation between Δ*K_SV_* and BMP concentration and fibril length is observed at pH 4.5 (Fig. 5*B*). Normalization of the % dye release per Δ*K_SV_* allows an approximate relative efficiency of membrane damage for samples of different type (monomer, fragmented and unfragmented fibrils) to be determined (Fig. 7). At pH 4.5, fragmented β_2_m fibrils give rise to an ∼2-fold greater increase in % dye release per Δ*K_SV_* than the equivalent unfragmented β_2_m fibrils for LUVs containing BMP. Additionally, for LUVs comprised of 50 mol % BMP, ∼2-fold more dye release per fibril-lipid interaction is observed for both fibrils types compared with LUVs comprising of only 12 mol % BMP. Increased fibril-induced membrane damage is unlikely to arise solely from a greater proportion of β_2_m fibrils interacting with the lipid bilayer at higher BMP concentrations. Thus, the difference in membrane damage efficiency observed for different lipid vesicle compositions, and fragmented versus unfragmented fibrils, either relates to a difference in susceptibility of particular membrane compositions to fibril-induced membrane damage, and/or a second activation step that requires specific fibril-lipid interactions.

The interaction between areas of positive charge localized on the β_2_m fibril surface/ends and negatively charge lipids may provide a rationale for the higher efficiency of membrane disruption observed at acidic pH for the POPG- and BMP-containing lipid mixtures compared with POPS-containing and zwitterionic lipid mixtures (Fig. 3*B*–*C*). The theoretical pI of β_2_m is 6.01 (Protparam, [59]). However, in a large fibril aggregate it is likely that significant charge-screening occurs within the protein core resulting in an altered pI and/or localized areas of surface charge. Although, the distribution of charge on the surface of the β_2_m fibrils is not currently known since an atomic resolution structure of these particles has not yet been determined, exposed areas of charge are more likely to be protonated at lower pH values. The presence of negatively charged groups on the lipid bilayer also attracts positively charged counterions, lowering the pH in the vicinity of the membrane interface compared with the bulk solution [60], [61] which may also facilitate protonation of the fibril surface. At pH 4.5 the serine head groups will be also protonated [62], whilst POPG and its structural isomer BMP are expected to remain negatively charged. Hence, the absence of dye release from POPS-containing and zwitterionic LUVs, and increase in β_2_m fibril-induced membrane damage typically observed for LUVs comprised of a greater proportion of POPG or BMP (Fig. 3*B*–*C*, also see Table S3), suggests that the interaction between positive charge on the β_2_m fibrils and the negative charge localized on the phospholipid backbone of POPG and BMP (Fig. 1*A*) results in membrane damage.

Although electrostatic interactions are likely to be important in the mechanism of β_2_m fibril-induced membrane damage, no simple relationship between the charge on the lipid head-group and the extent of fibril-induced membrane damage is observed. However, the pH of the solution also determines interfacial tension of the lipid vesicle, which in turn determines bilayer rigidity and, as a result, affects membrane stability [63]. Hence, the balance between β_2_m fibril and lipid electrostatic interactions is also likely to depend on the overall dynamics and structural properties of the membrane.

The structure of BMP is unusual from two perspectives; i) it is a phospholipid with two glycerol groups each with a single acyl chain, which results in a cone like shape in the lipid bilayer and ii) it possesses a unique *sn-*1 glycerophosphate backbone (typically associated with archeal glycerophospholipids), not the usual *sn-*3 stereochemistry of mammalian lipids [64]. Although BMP has been shown to mix with other phospholipids as a lipid, and not to act as a detergent as once speculated [56], [65], incorporation of BMP into zwitterionic lipid bilayers has been shown to induce less disorder in the liquid-crystalline phase, compared with the inclusion of equimolar concentrations of DOPG [65]. Altered disorder in BMP-containing membranes may account for the greater dye release observed upon interaction between β_2_m fibrils and BMP-containing liposomes when compared with POPG-containing LUVs. Additionally, due to the absence of a lipid ‘head-group’, BMP displays significant alkyl segments at the surface of the bilayer [56] which may decrease steric hindrance of the charged phospholipid backbone and/or form an additional binding platform for β_2_m fibril-lipid interactions.

Acyl chain length and saturation are key determinants of the dynamics and structural order of a lipid bilayer. However, it is difficult to disentangle the effect of acyl chain length/saturation and headgroup on the biophysical properties of BMP containing membranes compared with POPS- and POPG-containing LUVs due to the fundamental differences between the unusual structure of BMP and typical phosphatidyl lipid structures; whereby both lipid chains of BMP are directly attached to the phosphate backbone via individual glycerol moieties, rather than both chains arising from a single glycerol group as in phosphatidyl lipids (see Fig. 1*A*). The 18∶1 variant of BMP is typically the most common form of BMP found *in vivo* (although its contribution is lower in some cell types), with 18∶1 BMP comprising ∼57% of the total BMP isolated from human liver (see for example [66]–[69]). Hence, the 18∶1 form of BMP, its isomer 18∶1 POPG and equivalent 18∶1 POPS were utilized herein. Although both acyl chains of 18∶1 BMP are unsaturated, and 18∶1 POPS and POPG consist of one saturated and one unsaturated chain. However, no substantial difference in the efficiency of dye release was observed for POPS-containing LUVs compared with DOPS-containing vesicles (Fig. S5*B)*. Similarly, LUVs containing DOPG lipids give rise to similar (possibly slightly lower) extents of dye release than their POPG-containing counterparts (Fig. S5*C*). Hence, chain saturation effects do not appear to dominate the efficiency of dye release observed in BMP-containing vesicles.

Cholesterol has also been shown to influence membrane dynamics [70]. The general view is that cholesterol causes an increase in bilayer rigidity by reducing the number of trans-gauche isomerizations accessible to the acyl chains of the surrounding lipid molecules. However, it has also been shown that the of effect cholesterol on membrane structure depends on the lipid chain content, saturation and length [71], and the presence of cholesterol also induces negative spontaneous curvature [72] and so can contribute to the generation of negative membrane curvature stress thought to mediate membrane fusion events [73], [74] and the formation of toroidal lipid pores [75]. There is mounting evidence that the presence of cholesterol can also modulate membrane damage and cytotoxicity caused by amyloid proteins (see for example [76]–[79]). Incorporation of 25 mol % cholesterol into DOPS and DOPG LUVs has been shown previously to decrease membrane damage induced by fibrillar α-synuclein [13]. However, for fragmented β_2_m fibrils we observe approximately 4-fold more dye release from LUVs containing >15 mol % cholesterol compared with LUVs containing less cholesterol (Fig 4C). Hence, membrane damage caused by different amyloid proteins may not follow a simple trend, but involve specific interactions between each amyloid species and the lipid bilayer.

### Biological implications of the interaction between β_2_m fibrils and cellular membranes

Biological membranes enable regulation of the specific biochemical environments essential for cellular physiology [27]. BMP is virtually exclusive to the membranes of vesicles in the endocytic pathway. High concentrations of BMP are found in the membranes of late endosomes; wherein the concentration of BMP is approximately 15 mol %, but the inner membrane leaflet of late endosomes can comprise of as much as 70 mol % BMP [43], [44], [80]. Endosomal maturation is also associated with increased acidification of the vesicle interior resulting in a pH gradient across the membrane [81]. An analogous (but inversed) pH gradient is created using the dye release assay described here, with neutral pH in the vesicle interior and acidic pH (4.5–6.5) on the vesicle exterior. Thus, as endocytosed β_2_m fibrils are trafficked through the endocytic pathway to lysosomes [46], they are likely to encounter BMP-containing membranes under acidic conditions, comparable to the conditions shown to give rise to maximal dye release *in vitro* herein.

Although no direct evidence of gross disruption of endosomal vesicles or lysosomes upon interaction with β_2_m fibrils has been reported, other studies have shown that amyloid sequences can increase the permeability of lysosomal membranes and point to an increase in lysosomal membrane potential as a feature of several amyloid disorders, including Alzheimer’s and Parkinson’s disease [82]–[89]. The unusual structure and stereochemistry of BMP are thought to be responsible for important roles in endosomes including: structural integrity; endosome maturation; and protein, lipid and cholesterol sorting and trafficking [43], [80], [90], [91]. BMP is essential for invagination of the limiting membrane in endosomes, in which the physical properties of BMP may help stabilize the resultant small intraluminal vesicles [55], [56]. Disruption of this process or promotion of small vesicle formation may constitute a mechanism of fibril induced cellular damage. However, the mechanism of β_2_m fibril-induced membrane disruption and how this manifests *in vivo* remain unclear.

The results of this study provide a biophysical rationale for the possible involvement of BMP-containing vesicles of endosomal origin in the cellular mechanism of β_2_m cytotoxicity and resulting DRA pathology. Further investigation of the interaction between amyloid fibrils formed from different protein sequences and anionic lipid species associated with different intracellular membranes is warranted and may help elucidate the diverse pathologies associated with DRA and other amyloid diseases.

## Supporting Information

Figure S1 **Dye leakage following addition of β_2_m to POPC/cholesterol or POPG/cholesterol LUVs.** Dye release from LUVs consisting of (*A*) 75 mol % POPC: 25 mol % cholesterol or (*B*) 75 mol % POPG: 25 mol % cholesterol at pH 7.4, 37°C. Dye release from LUVs consisting of (*C*) 75 mol % POPC: 25 mol % cholesterol and (*C*) 75 mol % POPG: 25 mol % cholesterol at pH 4.5, 37°C. Dye release was measured 20 min (*solid bar*), or ≥2 h (*open bar*) after the addition of β_2_m monomer, fragmented or unfragmented fibrils. Error bars represent 1 S.D. of the mean from three replicates.
(TIF)

Figure S2 **DLS and cryo-EM characterization of LUVs comprised of POPC/cholesterol or POPG/cholesterol extruded at 400 nm.** All vesicles were extruded using a 400 nm membrane and washed in *Assay Buffer* at pH 7.4. For each lipid mixture, the DLS size distribution (*left*) and representative cryo-EM images are shown for vesicles resuspended in *Assay Buffer* at pH 4.5 or pH 7.4 (*right*). The DLS traces represent a histogram fit using the regularization method for a single run (pH 4.5, *red solid line*; pH 7.4, *blue dashed line*). Typically, three measurements were made from each sample. (*A*) LUVs comprised of 75 mol % POPC: 25 mol % cholesterol and (*B*) LUVs comprised of 75 mol % POPG: 25 mol % cholesterol. Scale bar 250 nm.
(TIF)

Figure S3 **DLS and cryo-EM characterization of LUVs containing BMP extruded at 400 nm.** All vesicles were extruded using a 400 nm membrane and washed in *Assay Buffer* at pH 7.4. For each lipid mixture, the DLS size distribution and representative cryo-EM images are shown for vesicles resuspended in *Assay Buffer* at pH 4.5 and pH 7.4. The DLS traces represent a histogram fit using the regularization method for a single run (pH 4.5, *red solid line*; pH 7.4, *blue dashed line*). Typically, three measurements were made from each sample. LUVs comprising of 36 POPC: 20 POPE: 7 SM: 25 cholesterol (mol/mol) (*A*) minus BMP, (B) plus 12 mol % BMP and (*C*) plus 50 mol %BMP. (*D*) LUVs comprised of 36 POPC: 20 POPE: 7 SM and 12 mol % BMP without cholesterol (i.e. the same lipid mixture as in (*B*) minus cholesterol). Scale bar 250 nm.
(TIF)

Figure S4 **DLS and cryo-EM characterization of LUVs containing 12 mol % BMP extruded at 100 nm.** Vesicles were extruded using a 100 nm membrane and washed in *Assay Buffer* at pH 7.4. The DLS size distribution and representative cryo-EM images are shown for LUVs comprised of 36 POPC: 20 POPE: 7 SM: 25 cholesterol (mol/mol) plus 12 mol % BMP (i.e. the same lipid mixture as in Fig. S3 *B*) resuspended in *Assay Buffer* at pH 4.5 or pH 7.4 (*right*). The DLS traces represent a histogram fit using the regularization method for a single run (pH 4.5, *red solid line*; pH 7.4, *blue dashed line*). Typically, three measurements were made from each sample. Scale bar 250 nm.
(TIF)

Figure S5 **Comparison of % dye release in the presence of fragmented fibrils for LUVs extruded at different sizes or containing lipids with the same head group but differently saturated acyl chains.** A) LUVs doped with 12 mol % BMP extruded at either 400 nm (data corresponding to Fig. 3, *solid bars*) or 100 nm (*open bars*). B) LUVs doped with either 12 mol % POPS (data corresponding to Fig. 3, *solid bars*) or 12 mol % DOPS (*open bars*). C) LUVs doped with either 12 mol % POPG (data corresponding to Fig. 3, *solid bars*) or 12 mol % DOPG (*open bars*). The remaining lipid for all LUVs comprised of 36 POPC: 20 POPE: 7 SM: 25 cholesterol (mol/mol). Dye release was measured 10 min after the addition of 6 µM monomer equivalent concentration of β_2_m fragmented fibrils to 5 µM lipid equivalent concentration of CF-loaded LUVs in *Assay Buffer* pH 4.5–7.4, 37°C. Error bars represent 1 S.E. from three independent repeats, each of three replicates (from Fig. 3) and 1 S.D. of the mean for three replicates for the other data shown here.
(TIF)

Figure S6 **Stern-Volmer plots of Trp fluorescence quenching of β_2_m monomers, fragmented and unfragmented fibrils in the absence of LUVs 10 min after the addition of LUVs comprising 0, 12 or 50 mol % BMP as in** **Fig. 5** **.** (*A*) pH 4.5 and (*B*) pH 7.4. β_2_m monomer (*blue*), fragmented (*red*) and unfragmented (*green*) fibrils in the absence (*closed symbols, solid line*) or presence of LUVs (*open symbols, dashed line*).
(TIF)

Figure S7 **Confocal microscopy of GUVs and β_2_m monomers.** (*A*) Confocal images of 80 mol % DOPC plus 20 mol % BMP GUVs in *Assay Buffer* at pH 7.4 in the absence of β_2_m protein. The control image is representative of all GUV compositions under the conditions tested. Confocal images of TMR-labeled β_2_m monomer incubated with DiD-labeled GUVs for 15 min at ambient temperature in (*B*) *Assay Buffer* at pH 7.4 or (*C*) *Assay Buffer* at pH 6.5. (*L-R*) TMR fluorescence (*red*), DiD-labeled GUVs (*green*), phase-contrast image soluble carboxyfluorescein added to vesicle exterior (*blue*) and intensity profile of TMR florescence across selected GUVs (*yellow line, B and C only*). Representative images for GUVs comprising (*i*) 100 mol % DOPC or (ii) 80 mol % DOPC plus 20 mol % BMP are shown for both pH values. Scale bar 10 µm.
(TIF)

Table S1 **Lipid composition in total mol % for the complex lipid mixes used to form LUVs herein i.e. 0, 12 or 50 mol % anionic lipid component with the remaining lipid made up of zwitterionic components in a mol/mol ratio of 36 POPC: 20 POPE: 7 SM: 25 cholesterol.**
(DOC)

Table S2 **Concentration of citric acid and sodium phosphate components in** ***Assay Buffer*** **prepared at pH** **4.5–7.4.** *Assay Buffer* consists of a total of a 50 mM mixture of citric acid and sodium phosphate plus 107 mM NaCl and 1 mM EDTA. This buffer enables buffering across a wide, physiologically relevant, pH range (4.5–7.4), whilst is also iso-osmotically balanced to the 50 mM sodium phosphate pH 7.4, 10 mM NaCl, 1 mM EDTA plus 50 mM CF on the LUV interior used for dye release experiments.
(DOC)

Table S3 **Percentage dye release corresponding to the data shown in** **Fig. 3** **.** LUVs comprised of 36 POPC: 20 POPE: 7 SM: 25 cholesterol (mol/mol) doped with 0, 12 or 50 mol % anionic lipid, POPS, POPG or BMP. Dye release was measured 10 min after the addition of 6 µM monomer equivalent concentration of β_2_m (*A*) monomers, (*B*) fragmented fibrils or (*C*) unfragmented fibrils to 5 µM equivalent lipid concentration of CF-loaded LUVs in *Assay Buffer* pH 4.5–7.4, 37°C. Different fonts represent the statistical significance of different experiments relative to that data obtained in 0 mol % anionic lipid^1–2^.
(DOC)

Table S4 **Hydrodynamic radii obtained by DLS for LUVs shown in Fig. S2–S4.**
(DOC)

Table S5 ***K_SV_*** **values (M^−1^) determined for quenching of Trp fluorescence for β_2_m monomers, fragmented and unfragmented fibrils in solution and 10 min after addition of LUVs comprised of 0, 12 or 50 mol % BMP, as in** **Fig. 5** **and Fig. S6.**
(DOC)
