# Supplementary figures and images for: β2-Microglobulin Amyloid Fibril-Induced Membrane Disruption Is Enhanced by Endosomal Lipids and Acidic pH
Source: PLoS One. 2014 Aug 6;9(8):e104492. doi: 10.1371/journal.pone.0104492 (PMC4123989; doi:10.1371/journal.pone.0104492)

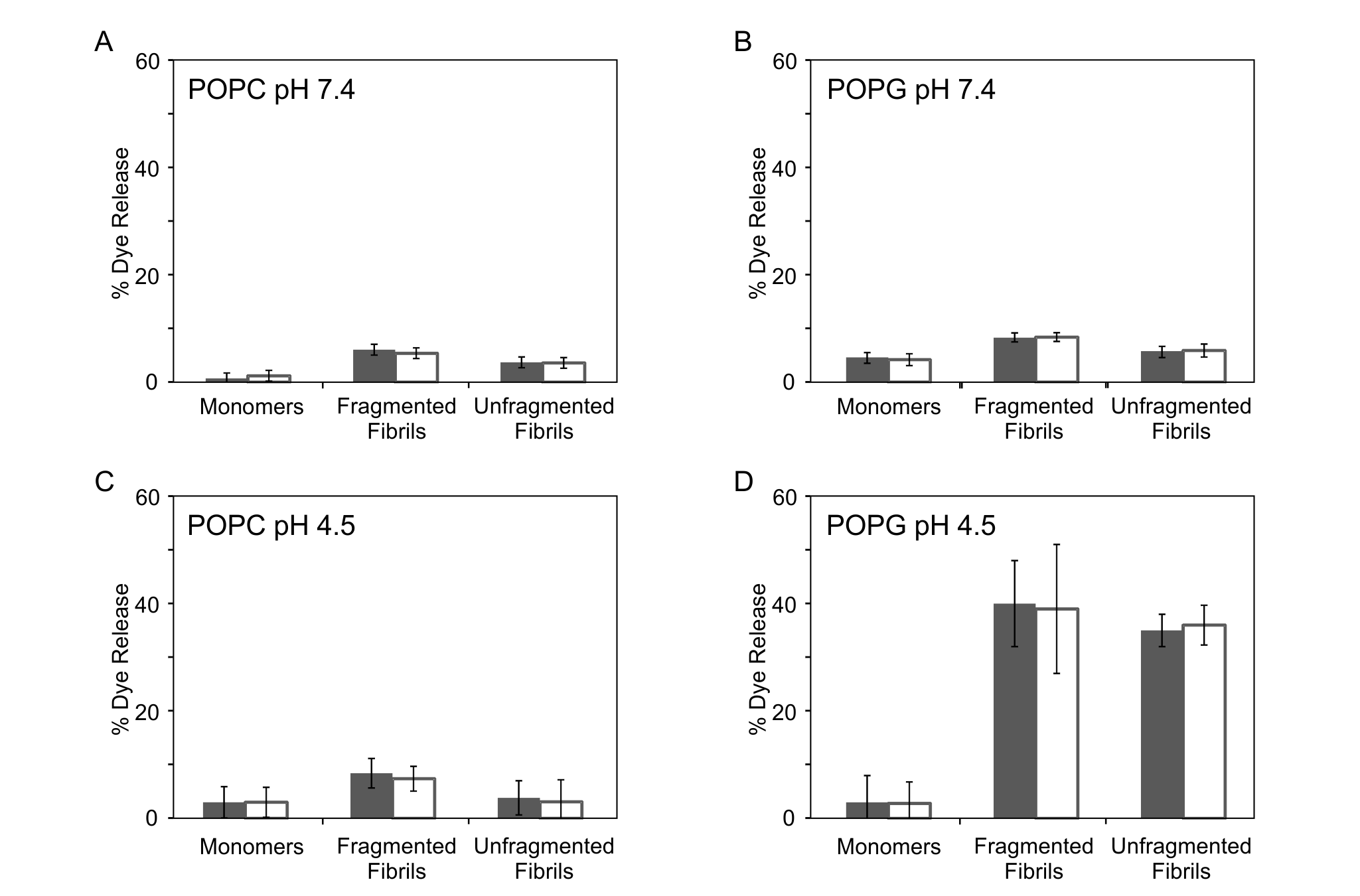

Supplement: Figure S1 — Dye leakage following addition of β2m to POPC/cholesterol or POPG/cholesterol LUVs. Dye release from LUVs consisting of (A) 75 mol % POPC: 25 mol % cholesterol or (B) 75 mol % POPG: 25 mol % cholesterol at pH 7.4, 37°C. Dye release from LUVs consisting of (C) 75 mol % POPC: 25 mol % cholesterol and (C) 75 mol % POPG: 25 mol % cholesterol at pH 4.5, 37°C. Dye release was measured 20 min (solid bar), or ≥2 h (open bar) after the addition of β2m monomer, fragmented or unfragmented fibrils. Error bars represent 1 S.D. of the mean from three replicates. (TIF) [file pone.0104492.s001.tif]

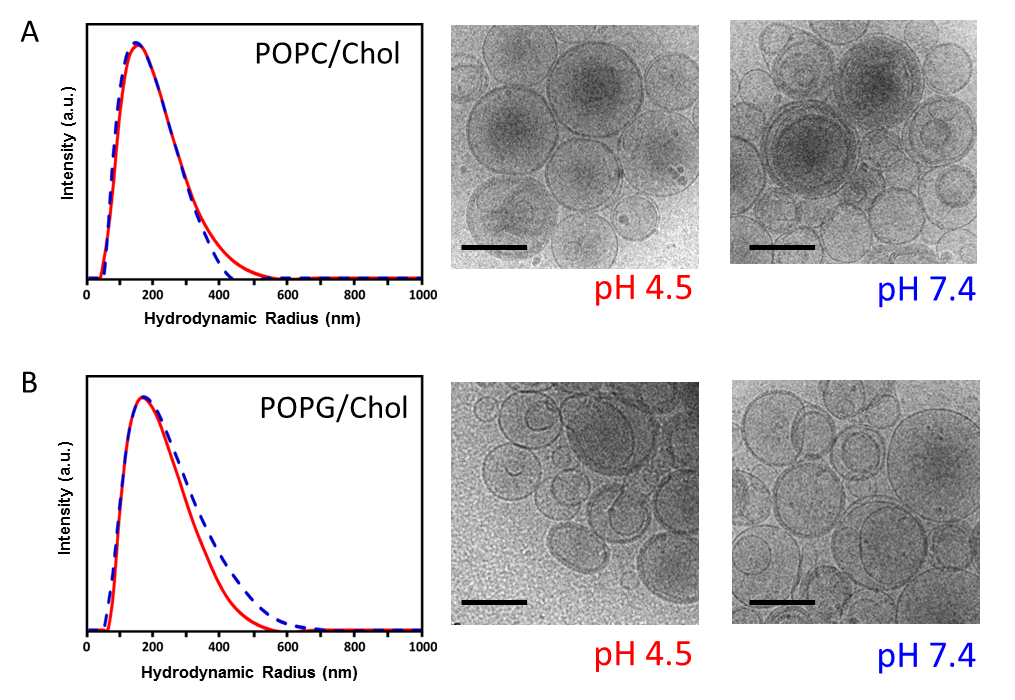

Supplement: Figure S2 — DLS and cryo-EM characterization of LUVs comprised of POPC/cholesterol or POPG/cholesterol extruded at 400 nm. All vesicles were extruded using a 400 nm membrane and washed in Assay Buffer at pH 7.4. For each lipid mixture, the DLS size distribution (left) and representative cryo-EM images are shown for vesicles resuspended in Assay Buffer at pH 4.5 or pH 7.4 (right). The DLS traces represent a histogram fit using the regularization method for a single run (pH 4.5, red solid line; pH 7.4, blue dashed line). Typically, three measurements were made from each sample. (A) LUVs comprised of 75 mol % POPC: 25 mol % cholesterol and (B) LUVs comprised of 75 mol % POPG: 25 mol % cholesterol. Scale bar 250 nm. (TIF) [file pone.0104492.s002.tif]

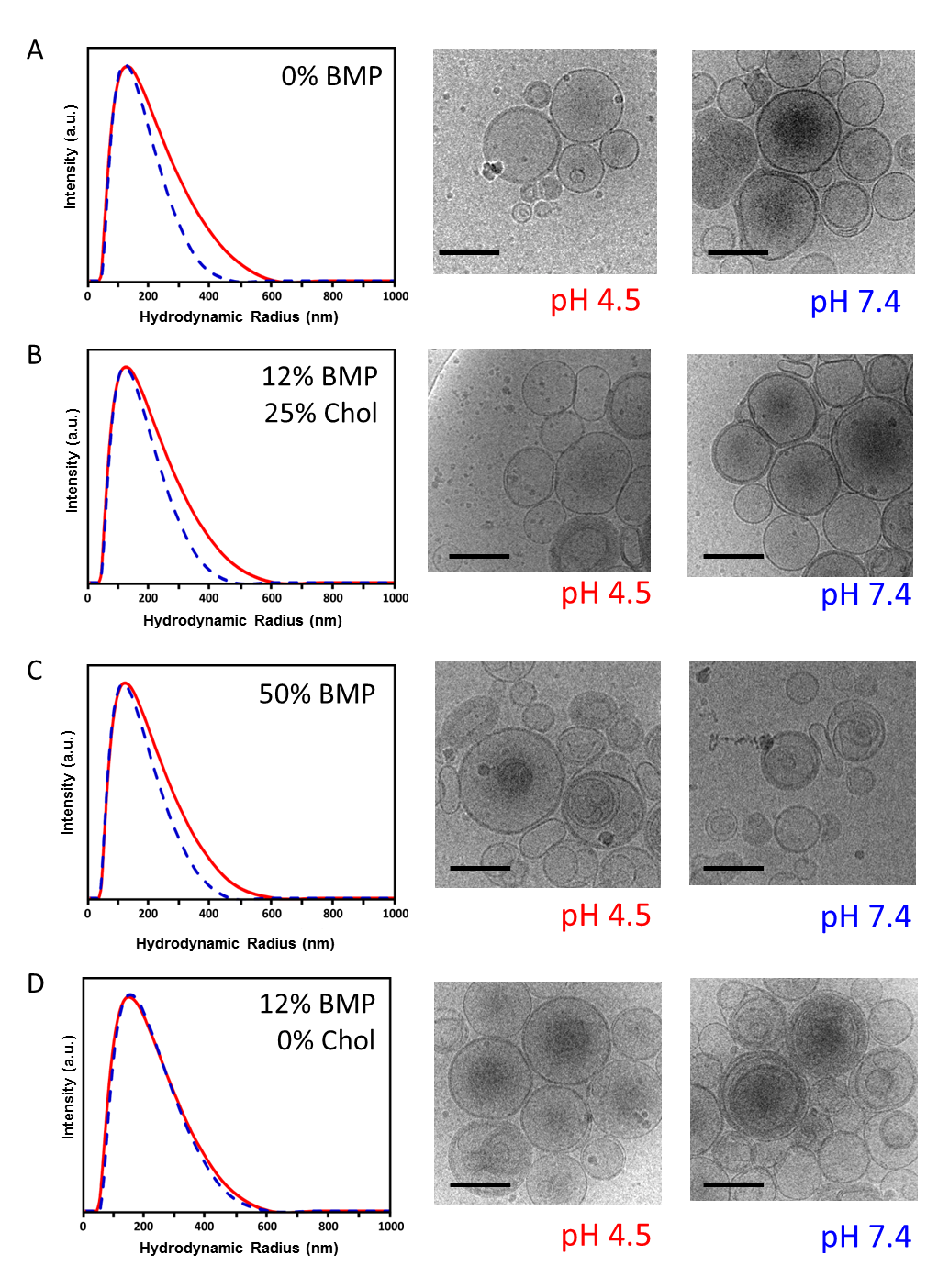

Supplement: Figure S3 — DLS and cryo-EM characterization of LUVs containing BMP extruded at 400 nm. All vesicles were extruded using a 400 nm membrane and washed in Assay Buffer at pH 7.4. For each lipid mixture, the DLS size distribution and representative cryo-EM images are shown for vesicles resuspended in Assay Buffer at pH 4.5 and pH 7.4. The DLS traces represent a histogram fit using the regularization method for a single run (pH 4.5, red solid line; pH 7.4, blue dashed line). Typically, three measurements were made from each sample. LUVs comprising of 36 POPC: 20 POPE: 7 SM: 25 cholesterol (mol/mol) (A) minus BMP, (B) plus 12 mol % BMP and (C) plus 50 mol %BMP. (D) LUVs comprised of 36 POPC: 20 POPE: 7 SM and 12 mol % BMP without cholesterol (i.e. the same lipid mixture as in (B) minus cholesterol). Scale bar 250 nm. (TIF) [file pone.0104492.s003.tif]

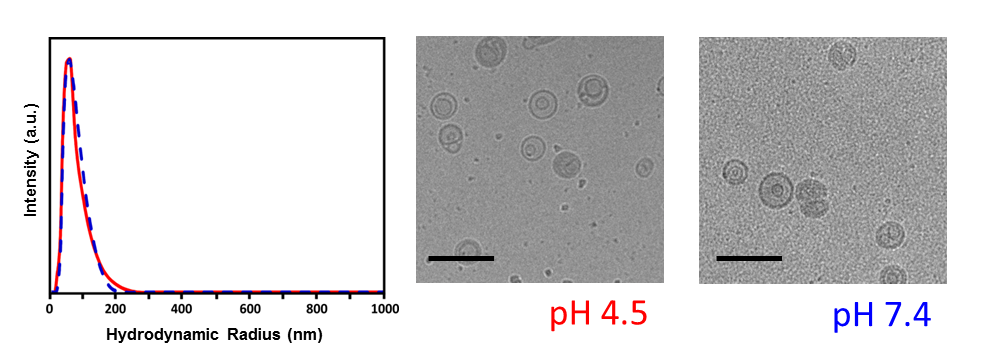

Supplement: Figure S4 — DLS and cryo-EM characterization of LUVs containing 12 mol % BMP extruded at 100 nm. Vesicles were extruded using a 100 nm membrane and washed in Assay Buffer at pH 7.4. The DLS size distribution and representative cryo-EM images are shown for LUVs comprised of 36 POPC: 20 POPE: 7 SM: 25 cholesterol (mol/mol) plus 12 mol % BMP (i.e. the same lipid mixture as in Fig. S3 B) resuspended in Assay Buffer at pH 4.5 or pH 7.4 (right). The DLS traces represent a histogram fit using the regularization method for a single run (pH 4.5, red solid line; pH 7.4, blue dashed line). Typically, three measurements were made from each sample. Scale bar 250 nm. (TIF) [file pone.0104492.s004.tif]

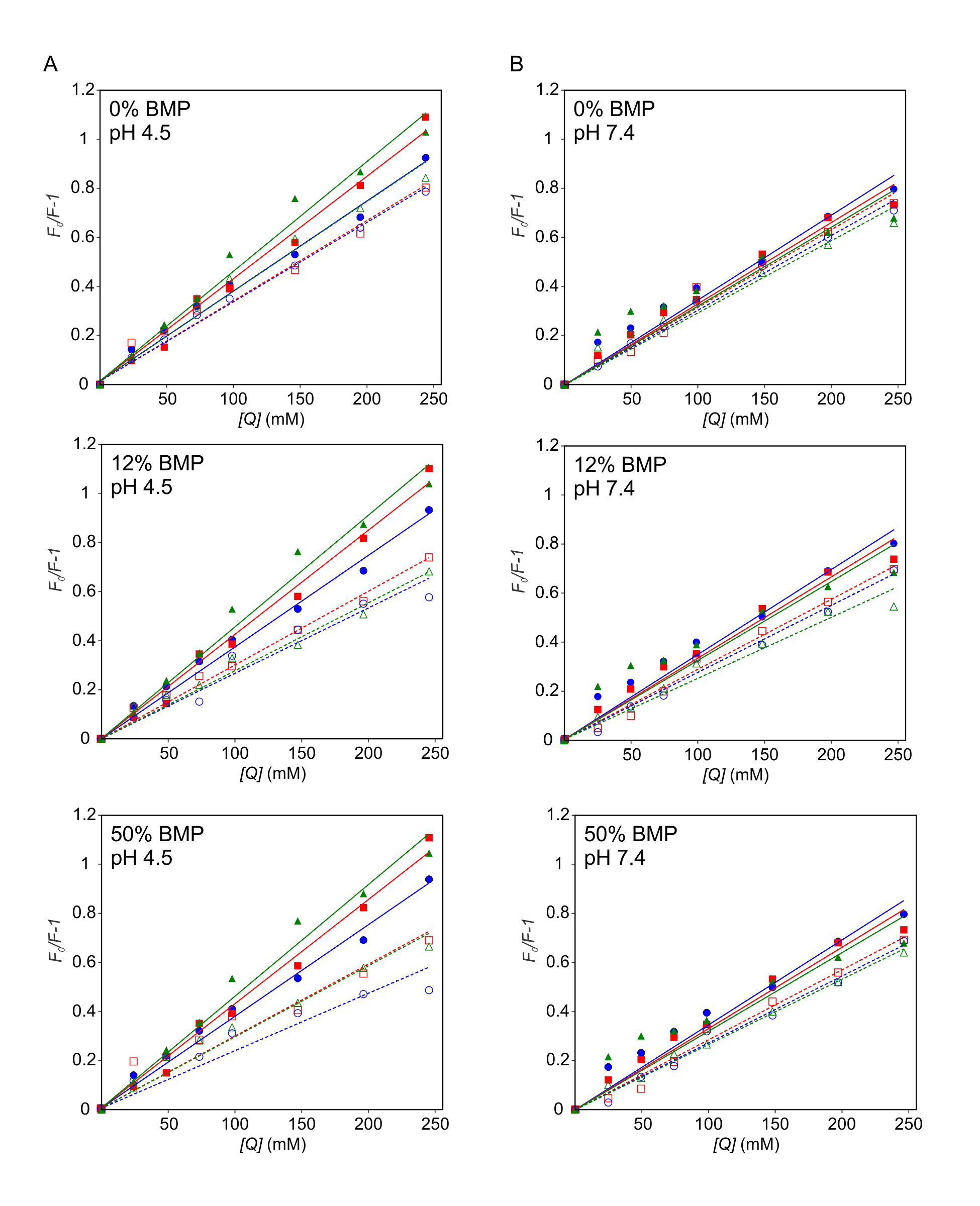

Supplement: Figure S6 — Stern-Volmer plots of Trp fluorescence quenching of β2m monomers, fragmented and unfragmented fibrils in the absence of LUVs 10 min after the addition of LUVs comprising 0, 12 or 50 mol % BMP as in Fig. 5 . (A) pH 4.5 and (B) pH 7.4. β2m monomer (blue), fragmented (red) and unfragmented (green) fibrils in the absence (closed symbols, solid line) or presence of LUVs (open symbols, dashed line). (TIF) [file pone.0104492.s006.tif]

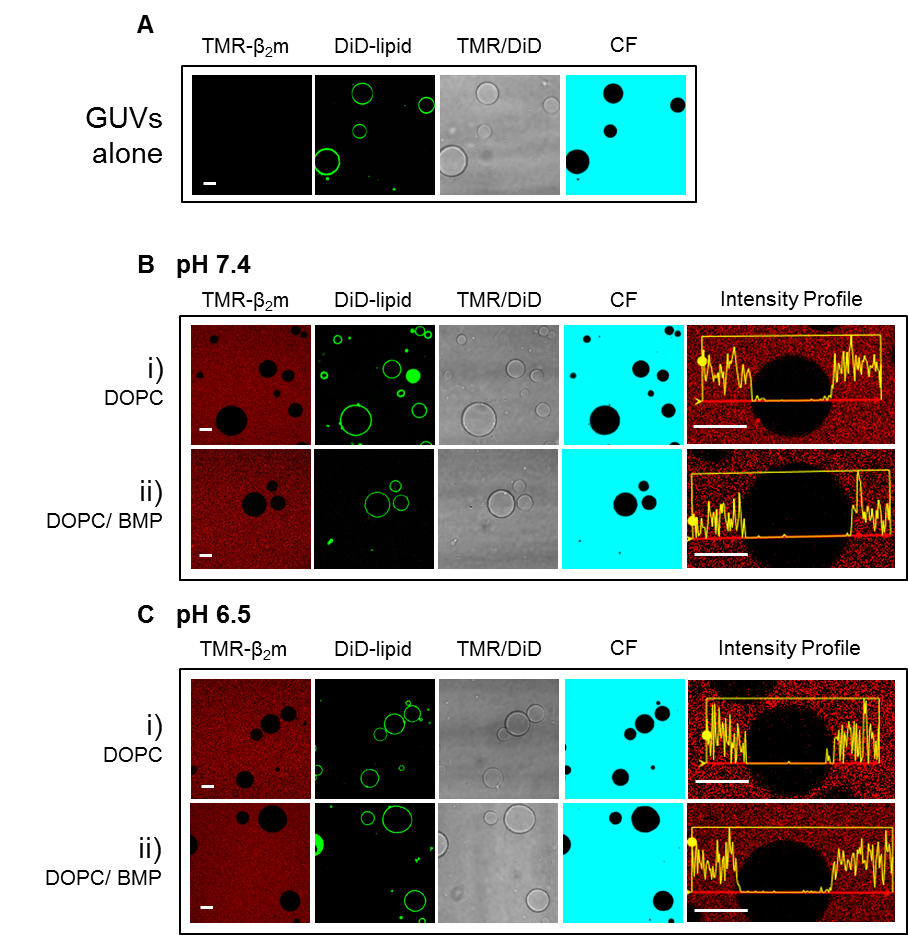

Supplement: Figure S7 — Confocal microscopy of GUVs and β2m monomers. (A) Confocal images of 80 mol % DOPC plus 20 mol % BMP GUVs in Assay Buffer at pH 7.4 in the absence of β2m protein. The control image is representative of all GUV compositions under the conditions tested. Confocal images of TMR-labeled β2m monomer incubated with DiD-labeled GUVs for 15 min at ambient temperature in (B) Assay Buffer at pH 7.4 or (C) Assay Buffer at pH 6.5. (L-R) TMR fluorescence (red), DiD-labeled GUVs (green), phase-contrast image soluble carboxyfluorescein added to vesicle exterior (blue) and intensity profile of TMR florescence across selected GUVs (yellow line, B and C only). Representative images for GUVs comprising (i) 100 mol % DOPC or (ii) 80 mol % DOPC plus 20 mol % BMP are shown for both pH values. Scale bar 10 µm. (TIF) [file pone.0104492.s007.tif]
