# Supplementary material for: β2-Microglobulin Amyloid Fibril-Induced Membrane Disruption Is Enhanced by Endosomal Lipids and Acidic pH
Source: PLoS One. 2014 Aug 6;9(8):e104492. doi: 10.1371/journal.pone.0104492 (PMC4123989; doi:10.1371/journal.pone.0104492)
Supplement: Table S1 — Lipid composition in total mol % for the complex lipid mixes used to form LUVs herein i.e. 0, 12 or 50 mol % anionic lipid component with the remaining lipid made up of zwitterionic components in a mol/mol ratio of 36 POPC: 20 POPE: 7 SM: 25 cholesterol. (DOC) [file pone.0104492.s008.doc]

| **Table S1. Lipid composition in total mol % for the complex lipid mixes used to form LUVs herein** i.e. 0, 12 or 50 mol % anionic lipid component with the remaining lipid made up of zwitterionic components in a mol/mol ratio of 36 POPC: 20 POPE: 7 SM: 25 cholesterol. | | | | |
| --- | --- | --- | --- | --- |
| **Anionic Lipid*^1^*** | **POPC (mol %)** | **POPE (mol %)** | **SM (mol %)** | **Cholesterol (mol %)** |
| **0 mol %** | 41 | 23 | 8 | 28 |
| **12 mol %** | 36 | 20 | 7 | 25 |
| **50 mol %** | 21 | 11 | 4 | 14 |
| *^1^ Anionic lipid component: POPG, POPS or BMP* | | | | |
