# Supplementary material for: β2-Microglobulin Amyloid Fibril-Induced Membrane Disruption Is Enhanced by Endosomal Lipids and Acidic pH
Source: PLoS One. 2014 Aug 6;9(8):e104492. doi: 10.1371/journal.pone.0104492 (PMC4123989; doi:10.1371/journal.pone.0104492)
Supplement: Table S2 — Concentration of citric acid and sodium phosphate components in Assay Buffer prepared at pH 4.5–7.4. Assay Buffer consists of a total of a 50 mM mixture of citric acid and sodium phosphate plus 107 mM NaCl and 1 mM EDTA. This buffer enables buffering across a wide, physiologically relevant, pH range (4.5–7.4), whilst is also iso-osmotically balanced to the 50 mM sodium phosphate pH 7.4, 10 mM NaCl, 1 mM EDTA plus 50 mM CF on the LUV interior used for dye release experiments. (DOC) [file pone.0104492.s009.doc]

| **Table S2**. **Concentration of citric acid and sodium phosphate components in *Assay Buffer* prepared at pH 4.5-7.4.** *Assay Buffer* consists of a total of a 50 mM mixture of citric acid and sodium phosphate plus 107 mM NaCl and 1 mM EDTA. This buffer enables buffering across a wide, physiologically relevant, pH range (4.5-7.4), whilst is also iso-osmotically balanced to the 50 mM sodium phosphate pH 7.4, 10 mM NaCl, 1 mM EDTA plus 50 mM CF on the LUV interior used for dye release experiments. | | | |
| --- | --- | --- | --- |
| **pH Buffered** | **[Citric Acid]**  **(mM)** | **[Na_2_HPO_4_]**  **(mM)** | **Ionic strength*^1^* of citrate and phosphate in Assay Buffer (mM)** |
| **pH 4.5** | 47 | 3 | 146 |
| **pH 5.5** | 41 | 9 | 137 |
| **pH 6.5** | 35 | 15 | 128 |
| **pH 7.4** | 31 | 19 | 122 |
| *^1^ Ionic strength calculated taking into account the activity coefficient of citrate and phosphate ions within Assay buffer* | | | |
