# Supplementary material for: β2-Microglobulin Amyloid Fibril-Induced Membrane Disruption Is Enhanced by Endosomal Lipids and Acidic pH
Source: PLoS One. 2014 Aug 6;9(8):e104492. doi: 10.1371/journal.pone.0104492 (PMC4123989; doi:10.1371/journal.pone.0104492)
Supplement: Table S3 — Percentage dye release corresponding to the data shown in Fig. 3 . LUVs comprised of 36 POPC: 20 POPE: 7 SM: 25 cholesterol (mol/mol) doped with 0, 12 or 50 mol % anionic lipid, POPS, POPG or BMP. Dye release was measured 10 min after the addition of 6 µM monomer equivalent concentration of β2m (A) monomers, (B) fragmented fibrils or (C) unfragmented fibrils to 5 µM equivalent lipid concentration of CF-loaded LUVs in Assay Buffer pH 4.5–7.4, 37°C. Different fonts represent the statistical significance of different experiments relative to that data obtained in 0 mol % anionic lipid1–2. (DOC) [file pone.0104492.s010.doc]

| **Table S3.** **Percentage dye release corresponding to the data shown in Fig. 3.** LUVs comprised of 36 POPC: 20 POPE: 7 SM: 25 cholesterol (mol/mol) doped with 0, 12 or 50 mol % anionic lipid, POPS, POPG or BMP. Dye release was measured 10 min after the addition of 6 µM monomer equivalent concentration of β_2_m (*A*) monomers, (*B*) fragmented fibrils or (*C*) unfragmented fibrils to 5 µM equivalent lipid concentration of CF-loaded LUVs in *Assay Buffer* pH 4.5-7.4, 37°C. Different fonts represent the statistical significance of different experiments relative to data obtained in 0 mol % anionic lipid^1-2^. | | | | |
| --- | --- | --- | --- | --- |
| **A. Monomer** | | | | |
| **Anionic Lipid component** | **pH 4.5 (%)** | **pH 5.5 (%)** | **pH 6.5 (%)** | **pH 7.4 (%)** |
| **0  mol %** | 3±2 | 2±2 | 2±3 | 1±3 |
| **12 mol % POPS** | 5±2 | 3±4 | 5±2 | 3±3 |
| **50 mol % POPS** | 4±3 | 3±4 | 2±2 | 2±3 |
| **12 mol % POPG** | 4±2 | 4±2 | 5±3 | 3±3 |
| **50 mol % POPG** | 5±2 | 6±4 | 7±4 | 4±2 |
| **12 mol % BMP** | 4±3 | 5±2 | 5±2 | 4±2 |
| **50 mol % BMP** | *9±3* | 8±3 | *10±3* | 8±3 |
| **B. Fragmented fibrils** | | | | |
| **Anionic Lipid component** | **pH 4.5 (%)** | **pH 5.5 (%)** | **pH 6.5 (%)** | **pH 7.4 (%)** |
| **0  mol %** | 8±2 | 9±4 | 6±3 | 2±2 |
| **12 mol % POPS** | 4±2 | 5±4 | 3±2 | 2±4 |
| **50 mol % POPS** | 7±4 | 5±5 | 6±2 | 3±4 |
| **12 mol % POPG** | ***13±3*** | ***1±5*** | ***16±2*** | 5±3 |
| **50 mol % POPG** | ***28±3*** | ***39±5*** | ***32±6*** | ***16±4*** |
| **12 mol % BMP** | ***19±5*** | ***18±3*** | ***17±4*** | 7±5 |
| **50 mol % BMP** | ***46±2*** | ***67±7*** | ***65±8*** | ***13±3*** |
| **C. Unfragmented Fibrils** | | | | |
| **Anionic Lipid component** | **pH 4.5 (%)** | **pH 5.5 (%)** | **pH 6.5 (%)** | **pH 7.4 (%)** |
| **0  mol %** | 4±5 | 5±4 | 2±6 | 2±4 |
| **12 mol % POPS** | 4±5 | 5±5 | 6±5 | 5±5 |
| **50 mol % POPS** | 4±5 | 3±4 | 2±5 | 3±5 |
| **12 mol % POPG** | 11±5 | 9±5 | 5±5 | 7±5 |
| **50 mol % POPG** | ***23±5*** | ***33±5*** | ***29±5*** | 12±5 |
| **12 mol % BMP** | *12±2* | 11±7 | 11±4 | 5±5 |
| **50 mol % BMP** | ***22±5*** | ***38±4*** | ***36±5*** | ***13±3*** |
| *^1^ Values shown in* ***bold italics*** *represent data with a P value of <0.05 and values shown in italics represent data with a P value of 0.05-0.1 calculated using the 2-tailed Student’s T-test assuming equal variance.*  *^2^ Error represents 1 S.E. from three independent repeats, each of three replicates* | | | | |
