# Supplementary material for: β2-Microglobulin Amyloid Fibril-Induced Membrane Disruption Is Enhanced by Endosomal Lipids and Acidic pH
Source: PLoS One. 2014 Aug 6;9(8):e104492. doi: 10.1371/journal.pone.0104492 (PMC4123989; doi:10.1371/journal.pone.0104492)
Supplement: Table S4 — Hydrodynamic radii obtained by DLS for LUVs shown in Fig. S2–S4. (DOC) [file pone.0104492.s011.doc]

| **Table S4. Hydrodynamic radii obtained by DLS for LUVs shown in Fig. S2-S4.** | | |
| --- | --- | --- |
| **Lipid Mixture** | **pH 4.5 (nm*^1^*)** | **pH 7.4 (nm)** |
| **POPC/cholesterol (Fig. S2*A*)** | 188±8 | 178±7 |
| **POPG/cholesterol (Fig. S2*B*)** | 221±8 | 229±5 |
| **0 mol % BMP**  **(Fig. S3*A*)** | 186±6 | 163±8 |
| **12 mol % BMP**  **(Fig. S3*B*)** | 168±8 | 160±5 |
| **50 mol % BMP**  **(Fig. S3*C*)** | 131±5 | 120±5 |
| **12 mol % BMP, 0 mol % cholesterol (Fig. S3*D*)** | 158±6 | 150±9 |
| **12 mol % BMP (100 nm extrusion) (Fig. S4)** | 56±8 | 58±5 |
| *^1^ Error reported as 1 S.D. from the weighted average of hydrodynamic radius determined from three runs for each sample* | | |
