# Supplementary material for: β2-Microglobulin Amyloid Fibril-Induced Membrane Disruption Is Enhanced by Endosomal Lipids and Acidic pH
Source: PLoS One. 2014 Aug 6;9(8):e104492. doi: 10.1371/journal.pone.0104492 (PMC4123989; doi:10.1371/journal.pone.0104492)
Supplement: Table S5 — KSV values (M−1) determined for quenching of Trp fluorescence for β2m monomers, fragmented and unfragmented fibrils in solution and 10 min after addition of LUVs comprised of 0, 12 or 50 mol % BMP, as in Fig. 5 and Fig. S6. (DOC) [file pone.0104492.s012.doc]

| **Table S5. *K_SV_* values (M^-1^) determined for quenching of Trp fluorescence for β_2_m monomers, fragmented and unfragmented fibrils in solution and 10 min after addition of LUVs comprised of 0, 12 or 50 mol % BMP, as in Fig. 5 and Fig. S6.** | | | | | | | | |
| --- | --- | --- | --- | --- | --- | --- | --- | --- |
|  | **pH 4.5** | | | | **pH 7.4** | | | |
|  | **No Lipid (M^-1^)** | **0 mol % BMP (M^-1^)** | **12 mol % BMP (M^-1^)** | **50 mol % BMP (M^-1^)** | **No Lipid (M^-1^)** | **0 mol % BMP (M^-1^)** | **12 mol % BMP (M^-1^)** | **50 mol % BMP (M^-1^)** |
| **Monomer** | 3.67±0.09 | 3.27±0.13 | 2.95±0.07 | 2.88±0.18 | 3.41±0.14 | 3.11±0.11 | 2.81±0.08 | 2.83±0.21 |
| **Fragmented** | 4.17±0.11 | 3.24±0.05 | 2.62±0.14 | 2.30±0.17 | 3.26±0.12 | 3.00±0.07 | 2.68±0.08 | 2.74±0.08 |
| **Unfragmented** | 4.47±0.14 | 3.66±0.12 | 2.72±0.11 | 2.85±0.12 | 3.16±0.25 | 2.89±0.15 | 2.45±0.11 | 2.62±0.06 |
| *Error represents 1 S.E. from linear regression* | | | | | | | | |
